# Dynamic Coassembly of Amphiphilic Block Copolymer and Polyoxometalates in Dual Solvent Systems: An Efficient Approach to Heteroatom-Doped Semiconductor Metal Oxides with Controllable Nanostructures

**DOI:** 10.1021/acscentsci.2c00784

**Published:** 2022-07-26

**Authors:** Yuan Ren, Wenhe Xie, Yanyan Li, Yuanyuan Cui, Chao Zeng, Kaiping Yuan, Limin Wu, Yonghui Deng

**Affiliations:** † Department of Chemistry, Department of Gastroenterology, Zhongshan Hospital of Fudan University, State Key Laboratory of Molecular Engineering of Polymers, Shanghai Key Laboratory of Molecular Catalysis and Innovative Materials, Fudan University, Shanghai 200433, P. R. China; ‡ Shimazu China Co LTD, Shanghai 200233, P. R. China; § School of Microelectronics, Fudan University, Shanghai 200433, P. R. China; ∥ Frontier Institute of Chip and System, State Key Laboratory of ASIC and System, Fudan University, Shanghai 200433, P. R. China; ⊥ Institute of Energy and Materials Chemistry, Inner Mongolia University, 235 West University Street, Hohhot 010021, P. R. China

## Abstract

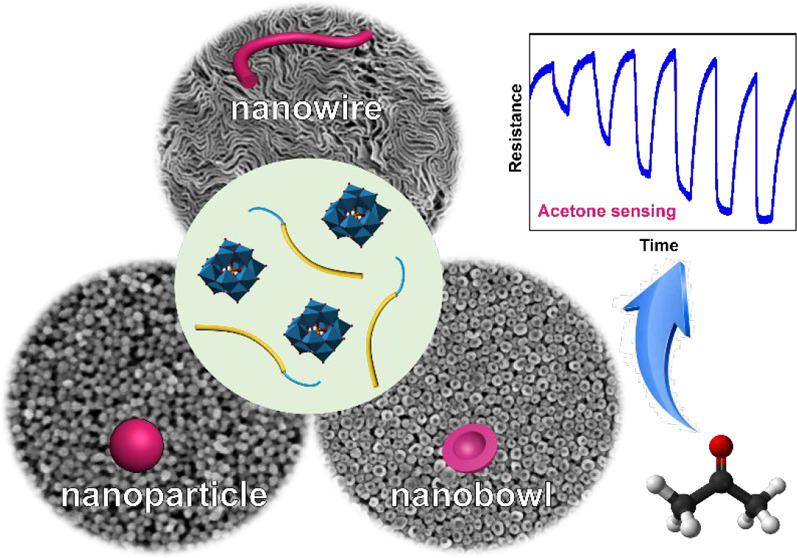

Dynamic coassembly of block copolymers (BCPs) with Keggin-type polyoxometalates (POMs) is developed to synthesize heteroatom-doped tungsten oxide with controllable nanostructures, including hollow hemispheres, nanoparticles, and nanowires. The versatile coassembly in dual *n-*hexane/THF solvent solution enables the fomation of poly(ethylene oxide)-*b*-polystyrene (PEO-*b*-PS)/POMs (*e.g*., silicotungstic acid, H_4_SiW_12_O_40_) nanocomposites with different morphologies such as spherical vesicles, inverse spherical micelles, and inverse cylindrical micelles, which can be readily converted into diverse nanostructured metal oxides with high surface area and unique properties via *in situ* thermal-induced structural evolution. For example, uniform silicon-doped WO_3_ (Si-WO_3_) hollow hemispheres derived from coassembly of PEO-*b*-PS with H_4_SiW_12_O_40_ were utilized to fabricate gas sensing devices which exhibit superior gas sensing performance toward acetone, thanks to the selective gas–solid interface catalytic reaction that induces resistance changes of the devices due to the high specific surface areas, abundant oxygen vacancies, and the Si-doping induced metastable *ε-*phase of WO_3_. Furthermore, density functional theory (DFT) calculation reveals the mechanism about the high sensitivity and selectivity of the gas sensors. On the basis of the as-fabricated devices, an integrated gas sensor module was constructed, which is capable of real-time monitoring the environmental acetone concentration and displaying relevant sensing results on a smart phone via Bluetooth communication.

## Abstract 2

A dynamic coassembly of block copolymers and polyoxometalates in mixed-solvent has been developed to synthesize heteroatom-doped metal oxides with controllable nanostructures and diverse compositions.

## Introduction

Amphiphilic block copolymers (BCPs) can self-assemble into a range of ordered mesostructures driven by the opposite long-range repulsive and short-range attractive forces because the constituent blocks are chemically incompatible but connected by covalent bonds.^1−6^ The self-assembled BCPs with specific compositions can be converted into ordered mesoporous materials (polymer, carbon, silica, *etc*.) by selectively removing some blocks via calcination or solvent extraction.^7−12^ This process can be viewed as self-templating synthesis. The coassembly of amphiphilic block copolymers (also serving as sacrificial templates) and inorganic precursors (*e.g*., metal alkoxides or salts, inorganic nanocrystals) with organic surfactants as sacrificial templates has proved to be an efficient route to produce ordered mesoporous materials with different compositions, high specific surface area, interconnected uniform pores, and rich active sites in the pore wall.^13−26^ Such a process is the well-known soft-templating synthesis. To endow ordered mesoporous materials with improved performance, great efforts have been made to introduce functional heteroatoms, nanoclusters, or even nanoparticles into the pore wall mostly via postmodification approaches; however, the post modification method usually leads to uncontrolled distribution of the guest species and pore blocking. To solve these problems, nanosized metal–oxygen clusters with intrinsic multiple compositions such as polyoxometalates (POMs) have recently been employed as precursor to synthesize mesostructures with different morphologies and heteroatom-doped frameworks without using other inorganic sources.^27−31^ For example, nanostructured inverse hexagonal polyoxometalate composite films were cast directly from solution using diblock copolymers poly(butadiene-*block*-2-(dimethylamino)ethyl methacrylate) (PB-*b*-PDMAEMA) as structure directing agents. Phosphomolybdic acid (H_3_[PMo_12_O_40_], H_3_PMo) as an inorganic source was selectively incorporated into the PDMAEMA domains because of electrostatic interactions between PMo^3–^ anions and protonated PDMAEMA.^27^ It is worth noting that polyoxometalates consist of transition metal elements (*e.g*., V, Mo, W) and nonmetallic element (*e.g*., Si, P); therefore, adopting POMs as the inorganic metal source to coassemble with amphiphilic block copolymers is particularly beneficial to the construction of multicomponent nanostructured metal oxides, carbides, and even nitrides.^32,33^ However, until now, little work has been done to the designed synthesis of nanostructured metal oxides with controllable morphology and chemical composition through coassembly of amphiphilic block copolymer with polyoxometalates. Our group recently succeeded in constructing 3D orthogonally cross-stacked metal oxide semiconducting nanowire arrays through the interfacial electrostatic coassembly of BCPs and POMs and micelle fusion and packing during solvent evaporation.^34,35^ However, previous studies were limited to the electrostatic interactions between block copolymers and polyoxometalates and rarely studied the phase transition behavior of block copolymers under the influence of solvents, so the structures obtained were monotonous (*e.g*., nanowires). The dynamic assembly of amphiphilic BCPs and POM clusters and corresponding derived controllable nanostructures have been rarely explored.

Herein, a novel dynamic interfacial coassembly method was developed to construct amphiphilic block copolymers/polyoxometalates nanocomposites in a mixed-solvent system consisting of tetrahydrofuran (THF) and *n*-hexane. Through tuning the volume ratio of THF/*n*-hexane, poly(ethylene oxide)-*b*-polystyrene (PEO-*b*-PS)/silicotungstic acid (H_4_SiW_12_O_40_) nanocomposites with diverse morphologies including spherical vesicles, inverse spherical micelles, and inverse cylindrical micelles could be obtained. Correspondingly, after thermal treatments, Si-doped WO_3_ nanostructures with different morphologies including nanobowls, nanoparticles, and nanowires were obtained. This general and flexible method could be extended to synthesize other nanostructured metal oxides (P-WO_3_, Si-MoO_3_, and P-MoO_3_) by coassembly of PEO-*b*-PS with other polyoxometalates (*e.g*., H_3_PW_12_O_40_, H_4_SiMo_12_O_40_, and H_3_PMo_12_O_40_) following a similar process. As an example, the Si-WO_3_ hollow hemispheres were used as semiconducting sensing materials to fabricate a gas sensor and showed excellent sensing performance to acetone, including high sensitivity (*R*_air_/*R*_gas_ = 37 vs 50 ppm), low limit of detection (<0.1 ppm), fast response speed (7 s), and good selectivity (*S*_acetone_*/S*_gas_ > 5), due to its high specific surface areas, highly crystalline framework, and abundant oxygen deficiency caused by homogeneous *in situ* Si-doping. Moreover, the gas sensing mechanism was systematically studied by *in situ* FTIR spectroscopy and theoretical DFT calculations. Furthermore, an integrated gas sensor module was fabricated, and real-time monitoring of acetone concentrations on a smart phone via Bluetooth communication was realized, which shows great potential in noninvasive early diagnosis of diabetes via detecting acetone in human exhaled breath.

## Results and Discussion

### Synthesis and Characterization of the Si-WO_3_ Nanobowls

**Scheme 1:**
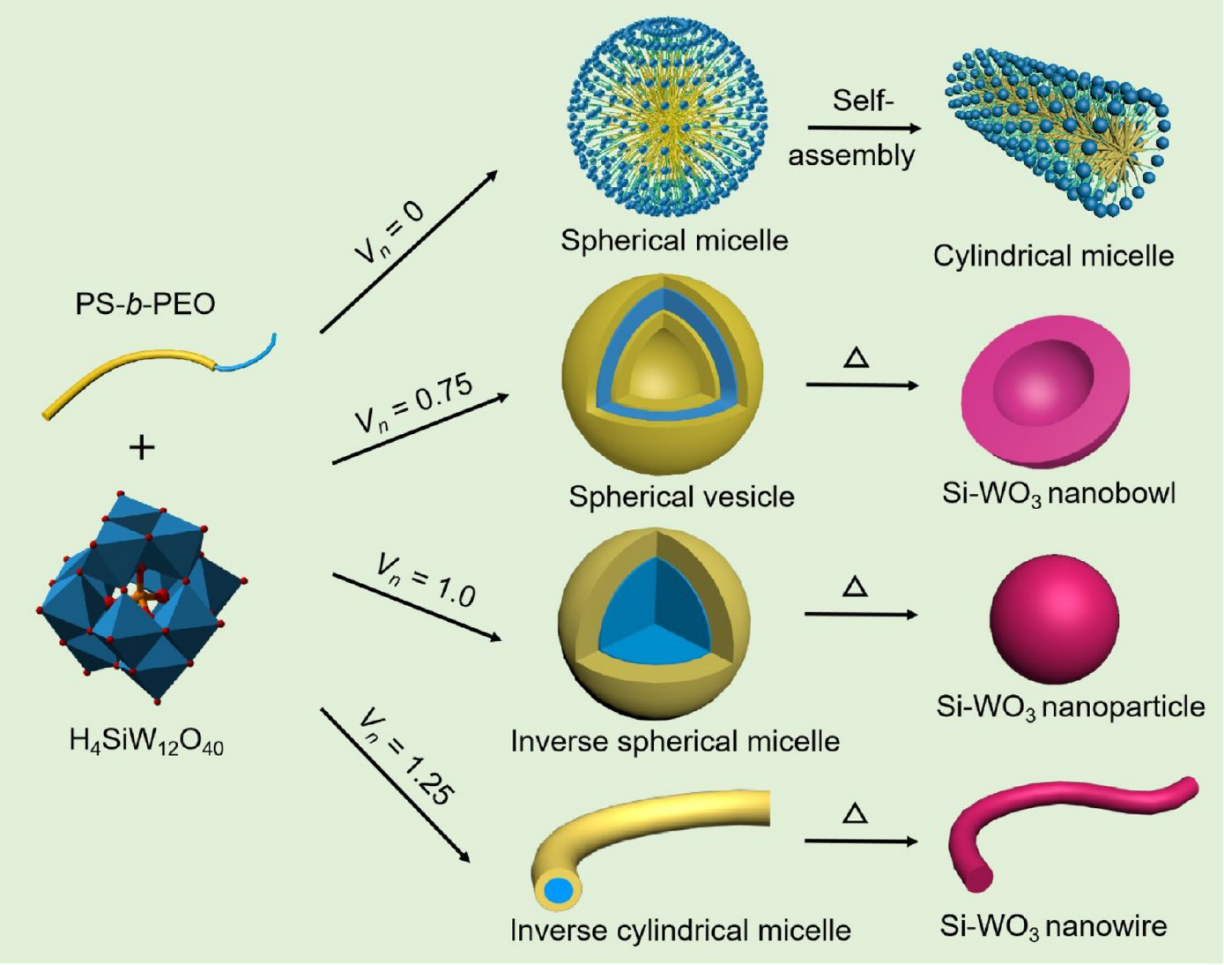
Coassembly of PEO-*b*-PS with H_4_SiW_12_O_40_ and Formation of PEO-*b*-PS/H_4_SiW_12_O_40_ Spherical Micelles, Spherical Vesicles, Inverse Spherical Micelles, and Inverse Cylindrical Micelles under Different Volume Ratio of *n*-Hexane/THF (*V*_*n*_*= V*_*n*-hexane_/*V*_THF_), and the Corresponding Derived Si-WO_3_ Nanostructures after Thermal Treatments

In this study, a mixed solvent consisting of miscible THF and *n*-hexane was employed to enable the coassembly of PEO-*b*-PS copolymers and polyoxometalates into various nanostructures by simply tuning the volume ratio of *n*-hexane/THF. Taking the assembly of PEO-*b*-PS copolymers and silicotungstic acid (H_4_SiW_12_O_40_, H_4_SiW) as a sample (Scheme 1), in THF solution without *n*-hexane, amphiphilic PEO-*b*-PS molecules interact with SiW_12_O_40_^4–^ to form core–shell spherical micelles with PS as the core and PEO/SiW_12_O_40_^4–^ as the shell, due to strong electrostatic attractions between the protonated PEO blocks (PEO-H^+^) and the SiW_12_O_40_^4–^ anions (Scheme 1a). When a certain amount of *n*-hexane, a poor solvent for SiW_12_O_40_^4–^, was added, the spherical micelles gradually transformed into spherical vesicles, inverse spherical micelles, and inverse cylindrical micelles, in sequence upon the increase of *n*-hexane due to the decreased solubility of POMs in the mixed solvent, and when these colloidal solutions containing PEO-*b*-PS/H_4_SiW_12_O_40_ micelles were cast onto the glass substrate, an organic–inorganic nanocomposite film could be obtained. The subsequent annealing treatments result in nanobowls, nanoparticles, and nanowires of Si-WO_3_, respectively, due to decomposition of PEO-*b*-PS and pyrolysis of H_4_SiW_12_O_40_ into Si-doped WO_3_ (Scheme 1).

**Figure 1:**
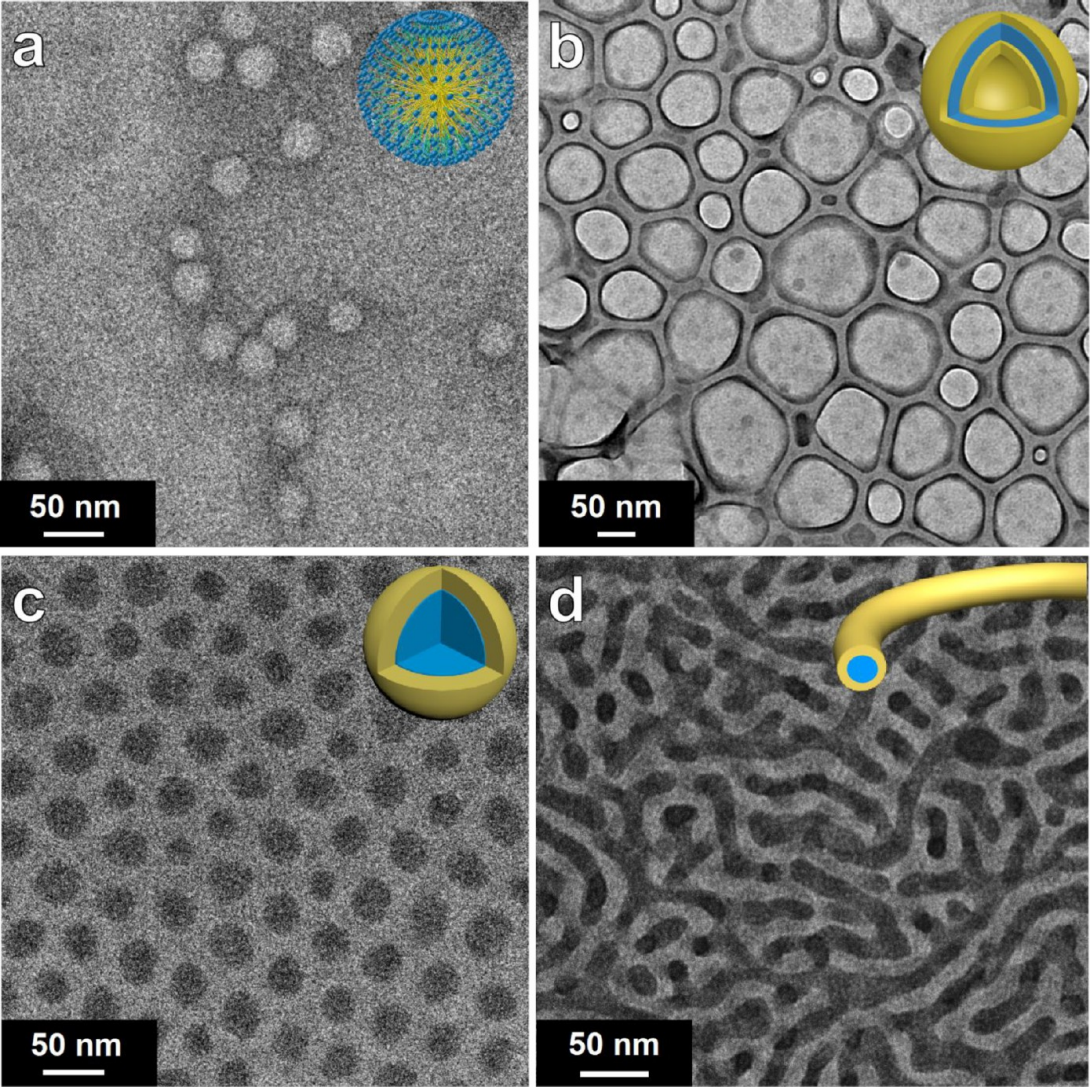
(a) Cryo-TEM and (b–d) TEM images and the corresponding structural models (insets) of the PEO-*b*-PS/H_4_SiW_12_O_40_ nanocomposites. (a) Spherical micelles (*V*_*n*-hexane_/*V*_THF_ = 0), (b) spherical vesicles (*V*_*n*-hexane_/*V*_THF_ = 0.75), (c) inverse spherical micelles (*V*_*n*-hexane_/*V*_THF_ = 1.0), and (d) inverse cylindrical micelles (*V*_*n*-hexane_/*V*_THF_ = 1.25).

Upon mixing PEO-*b*-PS and H_4_SiW_12_O_40_ hydrate, both predissolved in THF, a transparent light blue colloidal solution with a distinct Tyndall effect was formed immediately (Figure S2), indicating that PEO-*b*-PS/H_4_SiW_12_O_40_ hybrid micelles were formed directly. A cryogenic transmission electron microscopy (*Cryo*-TEM) image of the colloidal solution reveals the formation of core–shell spherical micelles with a gray PS chain as the core and PEO/SiW_12_O_40_^4–^ as the shell due to the dramatic mass contrast between the tungsten species and PS segments, and the average size of the spherical micelles was estimated to be about 35 nm (Figure 1a). Interestingly, when adding *n*-hexane into the colloid solution and the volume ratio of *n-*hexane/THF reaches 0.75, spherical vesicles with a diameter of about 80 ± 20 nm were obtained, with gray PS as the inner layer, dark PEO/H_4_SiW_12_O_40_ as the intermediate layer, and gray PS as the shell, as revealed by the TEM images (Figure 1b). With further increase of the volume ratio of *n*-hexane/THF from 0.75 to 1.0 and 1.25, the spherical vesicles gradually transformed into inverse spherical micelles (Figure 1c) and inverse cylindrical micelles (Figure 1d) respectively.

**Scheme 2:**
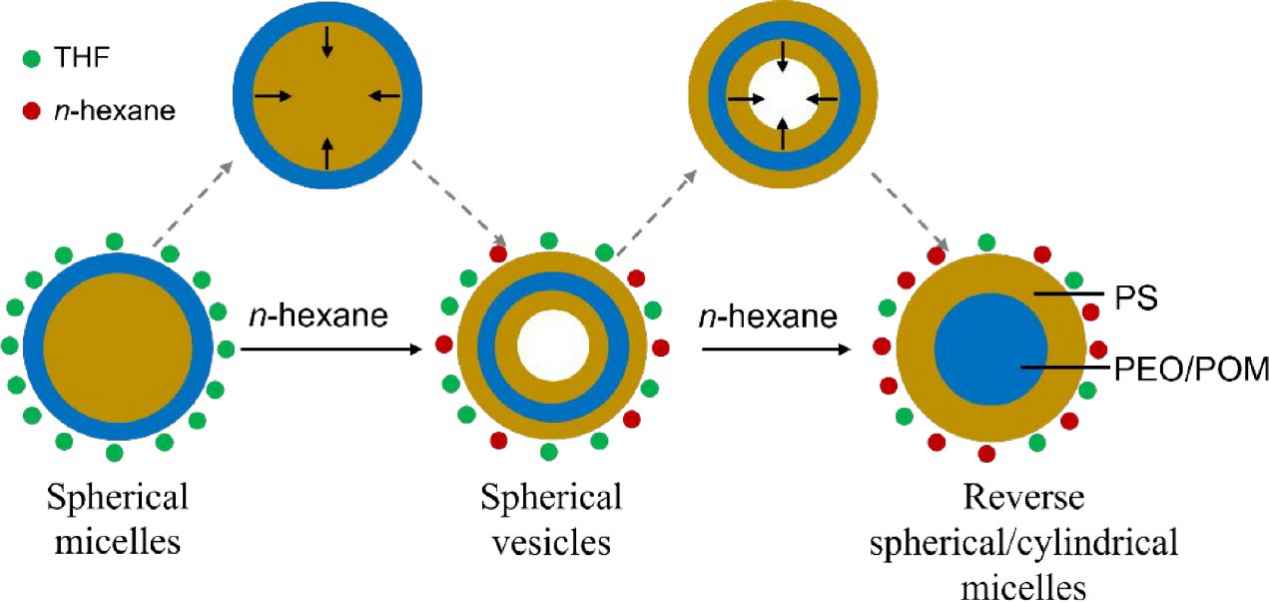
Illustration of the Transformation of the PEO-*b*-PS/H_4_SiW_12_O_40_ Hybrid Micelles/Vesicles under Different *n*-Hexane/THF Ratio

Such a morphology and structure evolution are mainly due to the decreasing solubility of the mixed solvents for PEO/SiW_12_O_40_^4–^. First, in the THF solution, driven by the microphase separation, spherical micelles with PS as the core and PEO/H_4_SiW_12_O_40_ as the shell can be formed to reduce the interface energy, due to the strong electrostatic Coulomb force (S^+^I^–^) between the protonated PEO block (PEO-H^+^) of PEO-*b*-PS surfactants and the inorganic POM anion (SiW_12_O_40_^4–^). With the addition of *n*-hexane, PS phase was swelled by *n*-hexane, while the PEO-H_4_SiW_12_O_40_ phase tends to shrink inward because *n*-hexane is a precipitating agent for PEO-H_4_SiW_12_O_40_. As a result, spherical vesicles with PS as the inner layer, PEO/H_4_SiW_12_O_40_ as the intermediate layer, and PS as the outer layer were formed. As more *n*-hexane was introduced in the assembly solution, the hydrophilic PEO-H_4_SiW_12_O_40_ phase shrinks further inward, and reverse spherical (or cylindrical) micelles with PEO/H_4_SiW_12_O_40_ as the core and PS as the shell were formed (Scheme 2).

The controllable formation of reverse spherical and cylindrical micelles was realized by tuning the BCPs/POMs mass ratio (Figure S1). The structures of self-assembled amphiphilic block copolymers are determined by the packing parameter: *p = v/a*_0_*l*_*c*_,^36−39^ where *v* and *l*_*c*_ represent the volume and length of the hydrophilic segment, respectively, while *a*_0_ denotes the contact area of the hydrophobic block. Generally, in the case of *p* < 1/3, spherical micelles or spheres with body-centered cubic (*bcc*) and face-centered cubic (*fcc*) packing modes can be formed. In the case of 1/3 < *p* < 1/2, cylindrical micelles or hexagonally packed cylinders (*Hex*) can be formed. In this study, when the weight ratio of BCPs/POMs reaches 1/3, the *p* value is less than 1/3, and spherical micelles were formed; and when the weight ratio of BCPs/POMs is 1/4, *p* value ranges between 1/3 and 1/2, namely, 1/3 < *p* < 1/2, and cylindrical micelles were formed. The structures of the above four kinds of micelles and vesicles were also be confirmed by dynamic light scattering (DLS) characterizations (Figure S2, S3).

**Figure 2:**
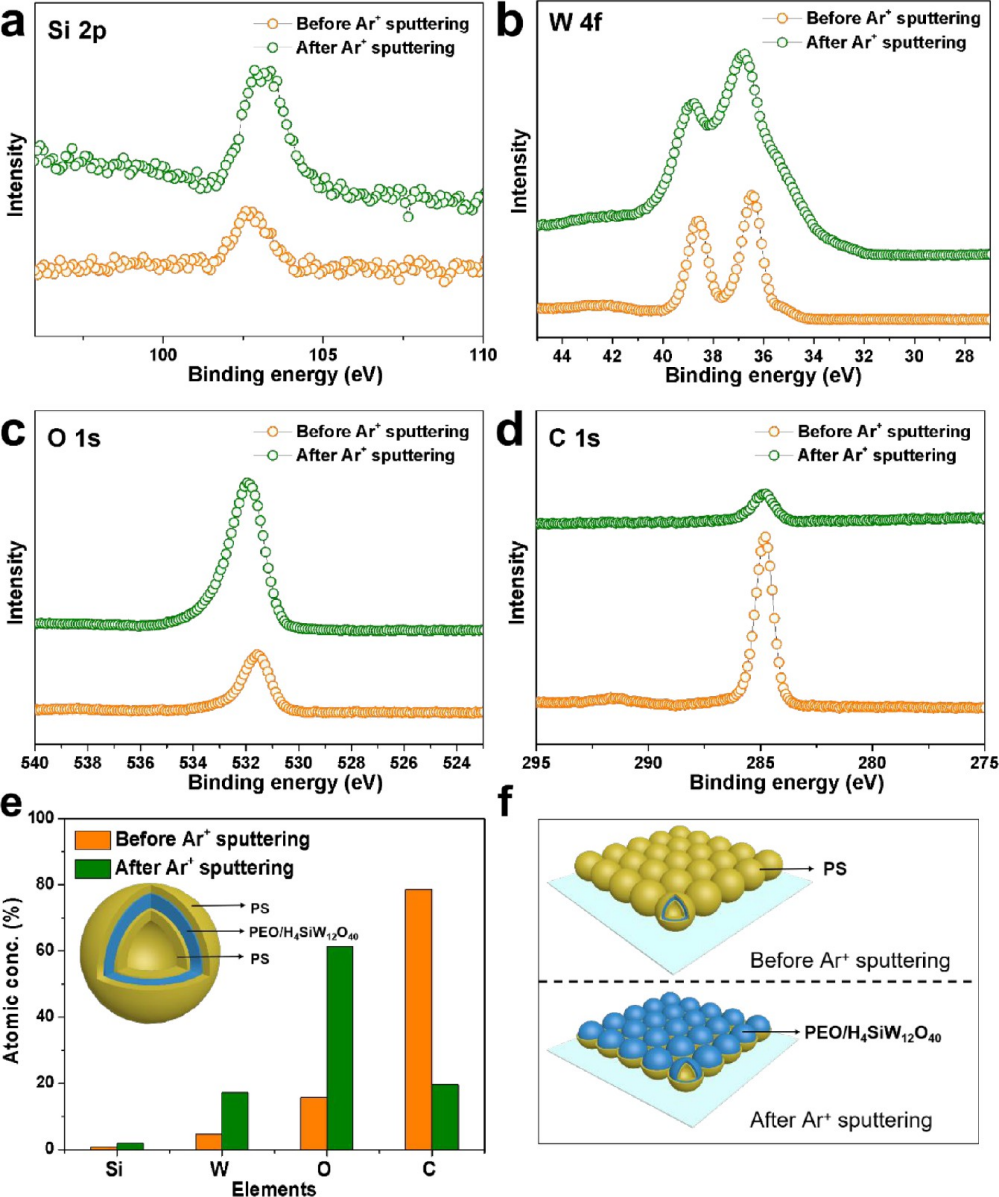
X-ray photoelectron spectroscopy (XPS) of PEO-*b*-PS/H_4_SiW_12_O_40_ spherical vesicles (*V*_*n*-hexane_/*V*_THF_ = 0.75) supported on silicon wafer before and after Ar^+^ sputtering: (a) Si 2p, (b) W 4f, (c) O 1s, and (d) C 1s. (e) Element contents measured by XPS and (f) the corresponding structural models of the PEO-*b*-PS/H_4_SiW_12_O_40_ spherical vesicles before and after Ar^+^ sputtering.

An *in situ* X-ray photoelectron spectroscopy (XPS) technique was employed to confirm the three-layered core-interlayer-shell structures of PEO-*b*-PS/H_4_SiW_12_O_40_ spherical vesicles. The PEO-*b*-PS/H_4_SiW_12_O_40_ nanocomposite film composed of spherical vesicles was fabricated on a silicon wafer (2 cm × 2 cm), and the element (Si, W, O, C) content was measured by XPS before and after the surface was sputtered using the Ar-GCIS beam, during which about 5–10 nm of surface coating would be stripped off (Figure 2). For the composite film composed of PEO-*b*-PS/H_4_SiW_12_O_40_ spherical vesicles, before Ar^+^ sputtering, the element content measured by XPS was Si = 0.83 at %, W = 4.70 at %, O = 15.80 at %, and C = 78.67 at %, respectively, indicating that the surface of hybrid film was mainly composed of organic copolymers. After Ar^+^ sputtering treatment for 180 s, the Si, W, and O content was increased to 1.97 at %, 17.17 at %, and 61.63 at %, respectively (Figure 2e). Correspondingly, the C content decreased to 19.55 at %. Extended delocalized electrons in aromatic rings can always result in satellite peaks, and their binding energy is several eV higher than the main sp^3^ C 1s peak. The π–π* satellite peak could be seen at ∼291.6 eV before etching but vanished after etching; it demonstrates that the PS phase dominates in the composite film surface. The above results indicate that the PEO/SiW_12_O_40_^4–^ layer was exposed to the surface after Ar^+^ sputtering, during which the PS domain in the surface of the composite film was etched away (Figure 2f). The combined results of TEM characterization and XPS analysis unambiguously confirmed the spherical vesicle structure with PEO/SiW_12_O_40_^4–^ acting as the wall and PS chains spreading outward and inward, respectively. In contrast, no π–π* satellite peak in the C 1s peak was observed for the cylindrical micelles (transformed from spherical micelles) before Ar^+^ sputtering (Figure S5a), and the W^4+^ species were found after Ar^+^ sputtering (Figure S4), indicating a different core–shell structure (PS as the core and PEO/SiW_12_O_40_^4–^ as the shell).

**Figure 3:**
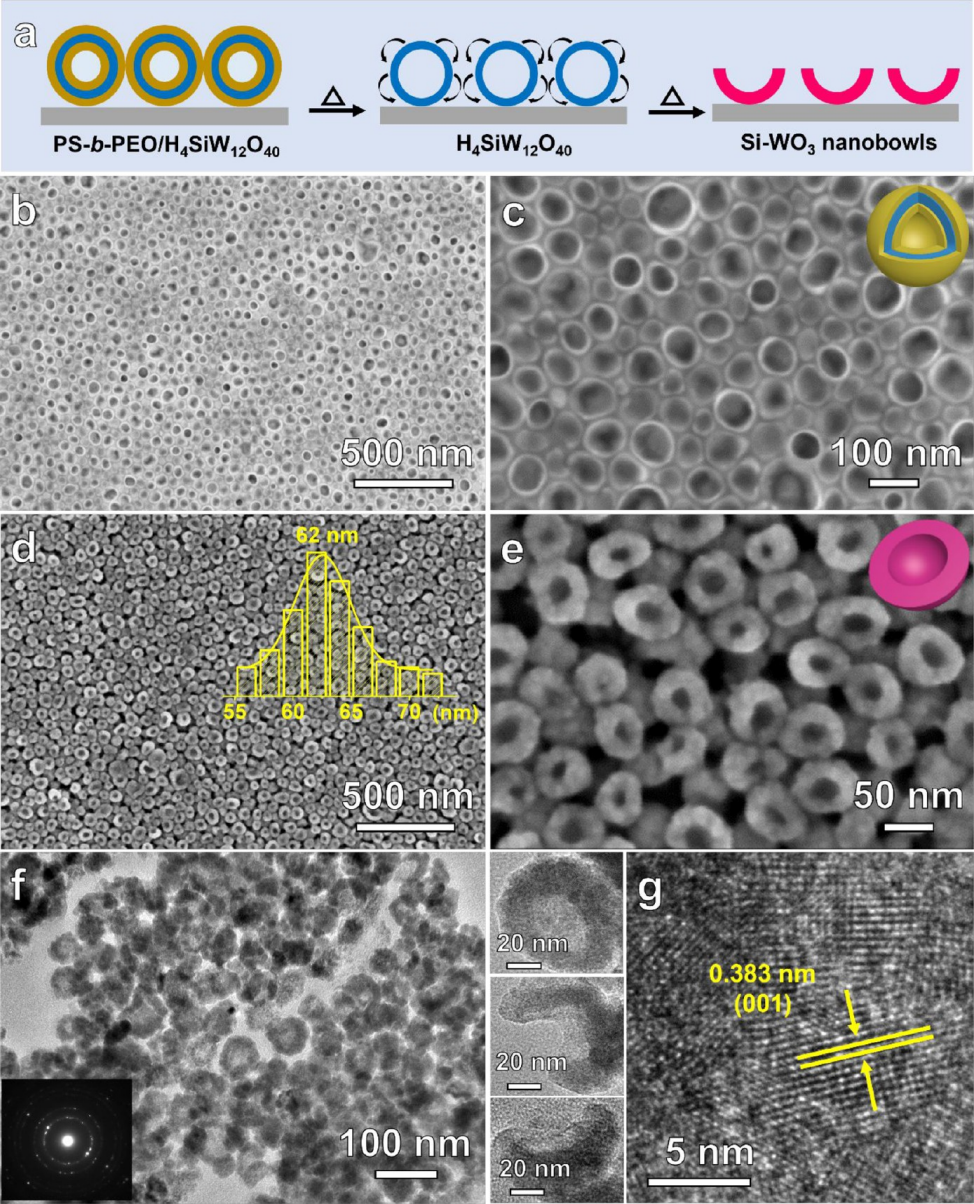
(a) Schematic illustration of the thermal-induced structural transformation of PEO-*b*-PS/H_4_SiW_12_O_40_ spherical vesicles into Si-WO_3_ nanobowls, (b, c) FESEM images of PEO-*b*-PS/H_4_SiW_12_O_40_ vesicles (*V*_*n*-hexane_*/V*_THF_ = 0.75) supported on silicon wafer, (d, e) FESEM, (f) TEM and SAED pattern (inset), and (g) HRTEM images of the Si-WO_3_ nanobowls. The insets in (c) and (e) are the corresponding structural models. The inset in (d) is diameter distribution of the Si-WO_3_ nanobowls.

Interestingly, PEO-*b*-PS/H_4_SiW_12_O_40_ spherical vesicles nanocomposite film deposited on the substrate underwent an unconventional thermal-induced structural transformation into Si-WO_3_ hollow hemispheres (nanobowls). In other words, the H_4_SiW_12_O_40_ species on the top of the spherical vesicles exposed larger surface areas and possess higher energy, which is relatively unstable. During the annealing process to decompose PEO-*b-*PS copolymers and pyrolyze H_4_SiW_12_O_40_ into Si-WO_3_, the H_4_SiW_12_O_40_ species would migrate to the contacting regions of neighboring vesicles and spaces between the vesicles and the substrate, thus forming Si-WO_3_ nanostructures of hollow hemispheres (Figure 3a). After casting the colloidal solution containing PEO-*b*-PS/H_4_SiW_12_O_40_ spherical vesicles onto the glass substrate, an inorganic–organic nanocomposite film was obtained after the solvent evaporates completely (Figure 3b, c). The subsequent thermal treatment at 500 °C in the N_2_ atmosphere and 400 °C in air can remove the organic template and decompose H_4_SiW_12_O_40_, forming unique nanobowl-like Si-doped WO_3_ nanoparticles. FESEM images reveal the Si-doped WO_3_ nanobowl-like particles are hollow hemispheres (HHSs) structures of Si-WO_3_ with an inner diameter of about 40 nm, external diameter of about 62 nm, and thickness of about 11 nm (Figure 3d, e). TEM characterizations at low magnification and from different directions confirmed unusual “nanobowls” morphology of Si-WO_3_, and the selected area electron diffraction (SAED) patterns with spotty rings indicate a polycrystalline property of the framework (Figure 3f, S6). A high-resolution TEM (HRTEM) image showed the lattice spacing of 0.383 nm, corresponding to the (001) plane of orthorhombic WO_3_ (JCPDS. 20-1324) (Figure 3g). Energy dispersive spectra (EDS) show a homogeneous distribution of the W, O, Si element throughout the Si-WO_3_ HHSs sample (Figure S7). The abnormally high Si content is due to the overlap of the peaks of W and Si elements. In this case, it is difficult to evidence Si at low concentrations by EDS when the W is present at high concentration because all energies of the SiK and WM are very close.^44−47^

In order to investigate the structural transformation process, the nanocomposite film composed of PEO-*b*-PS/POMs spherical vesicles was, respectively, treated at 500 °C for 0.5, 1.0, and 2.0 h (Figure S8). Before annealing, they possess spherical morphology (Figure 3a, b). After annealing at 500 °C for 0.5 h, due to the gradual decomposition of block copolymers, H_4_SiW_12_O_40_ species anchored at the micelles’ surface can gradually migrate from top of the vesicles to the contacting region between vesicles and substrate because they exposed a higher surface area and possess a high surface energy. At the same time, they were decomposed into Si-doped WO_3_, and nanobowls with thin walls and small openings were obtained. After annealing at 500 °C for 1 h, the tungsten species further migrate from the top to the contact region between the nanobowls and the substrate, resulting in nanobowls with thicker walls and larger openings; prolonging the annealing time to 2 h caused the nanobowls to become more flat and thicker due to the further migration of the tungsten species. The migration of tungsten species and the morphology evolution indicate that it is a thermal-induced structural transformation process.

It is worth mentioning that the morphology of the Si-WO_3_ nanobowls could be well controlled by fine-tuning the volume ratio of *n-*hexane/THF (*V*_*n*-hexane_*/V*_THF_) (Figure S9). The size of inner and outer diameters and wall thickness of Si-WO_3_ nanobowls are all controllable by tailoring *V*_*n*-hexane_*/V*_*THF*_ from 0.25 to 0.90. As increasing the *V*_*n*-hexane_*/V*_THF_ value, the volume of the inner cavity becomes smaller and the wall becomes thicker. The morphological changes of Si-WO_3_ nanobowls originate from the inward contraction of the PEO/H_4_SiW_12_O_40_ domain of the PEO-*b*-PS/H_4_SiW_12_O_40_ spherical vesicles because of the decrease of solubility for H_4_SiW_12_O_40_ in *n*-hexane/THF. Finally, when *V*_*n*-hexane_*/V*_THF_ reaches 1.0, the inner PS chains of the PEO-*b*-PS/H_4_SiW_12_O_40_ spherical vesicles completely disappeared, and the spherical vesicles evolved into inverse spherical micelles completely with PEO/H_4_SiW_12_O_40_ as the core and PS chain as the shell. Correspondingly, uniform solid Si-WO_3_ nanoparticles were obtained (Figures S12, S13).

**Figure 4:**
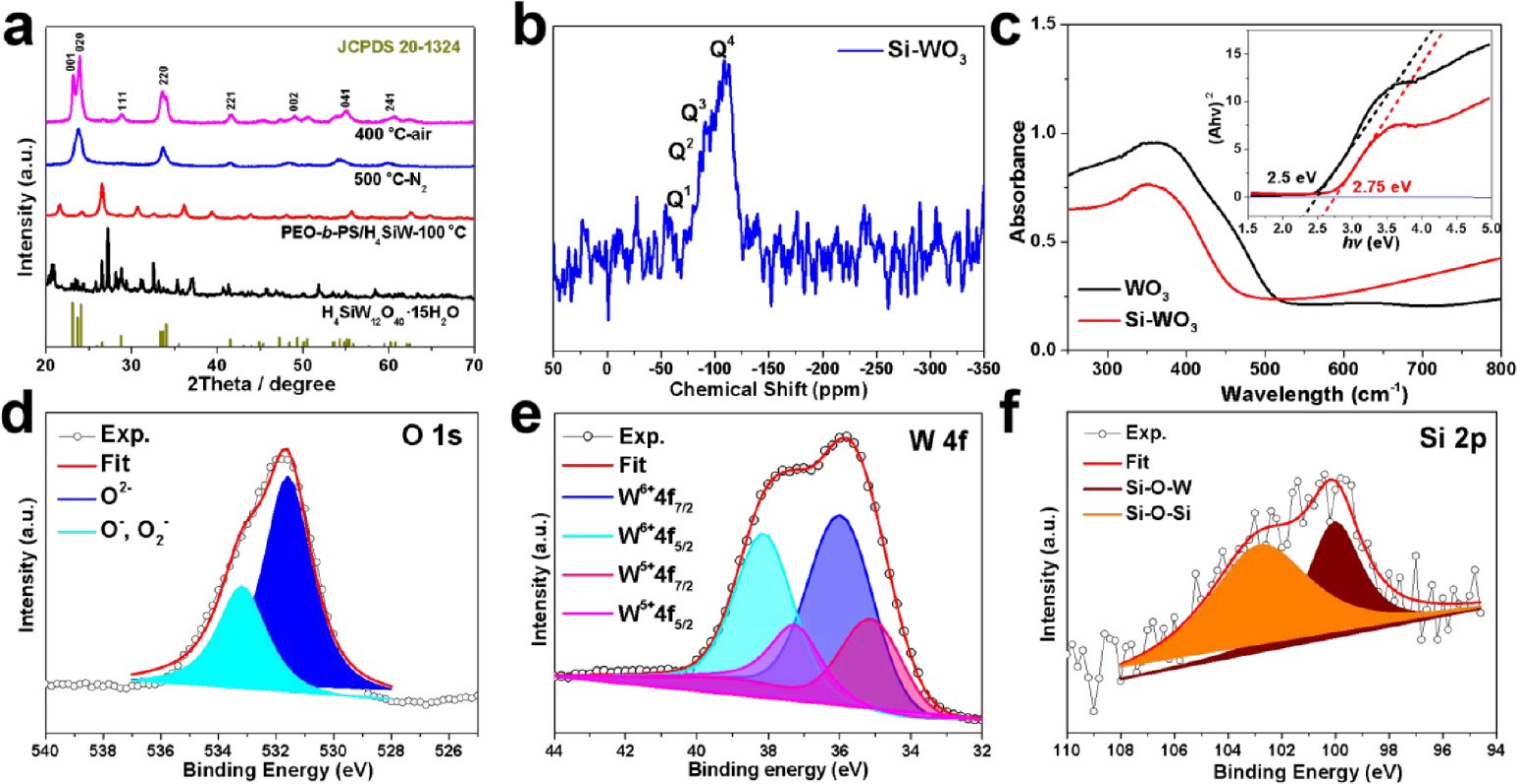
(a) XRD patterns of the commercial H_4_SiW_12_O_40_·15H_2_O, PEO-*b*-PS/H_4_SiW_12_O_40_ nanocomposites treated at 100 °C, and Si-WO_3_ hollow hemispheres obtained after thermal treatment at 500 °C in N_2_ and 400 °C in air. (b) Solid-state ^29^Si NMR spectrum of Si-WO_3_ hollow hemispheres; (c) UV–vis absorption spectra and the extracted band gap profile of the mesoporous WO_3_ obtained using WCl_6_ as the inorganic precursor and the Si-WO_3_ hollow hemispheres. X-ray photoelectron spectroscopy showing the (d) O 1s, (e) W 4f, and (f) Si 2p core level peak regions of Si-WO_3_ hollow hemispheres.

The X-ray diffraction (XRD) patterns of the PEO-*b*-PS/H_4_SiW_12_O_40_ nanocomposites and Si-WO_3_ hollow hemispheres are shown in Figure 4a; after pyrolysis in 500 °C-N_2_ and 400 °C-air, the H_4_SiW_12_O_40_ are completely transformed into Si-doped tungsten oxides, and diffraction peaks ranging from 0 to 70° matched well with the orthorhombic *ε-*WO_3_ phase (JCPDS no. 20-1324). The sharp diffraction peaks indicate a highly crystalline framework of Si-WO_3_ HHSs, and the average crystallite size is calculated to be 10.4 nm according to the Scherrer equation (*D* = *K*λ/*B* cos θ). The Fourier transform infrared (FTIR) characterizations also revealed the conversion of H_4_SiW_12_O_40_ into Si-WO_3_ (Figure S10). Before calcination, typical absorption peaks at 2922 and 3026 cm^–1^ can be clearly visible in the as-made PEO-*b*-PS/H_4_SiW_12_O_40_ composite, which was attributed to the —C–H and =C–H groups, respectively, and the absorption peaks at 788, 926, and 980 cm^–1^ were atrributed to Keggin polyoxometallates. While after calcination at 500 °C in N_2_ and 400 °C in air, all these peaks disappear, and a new absorption peak at 808 cm^–1^ appears, implying the formation of WO_3_ and a complete removal of the PEO-*b*-PS copolymers. The nitrogen adsorption–desorption isotherms of the Si-WO_3_ HHSs show a typical IV-type hysteresis loop, indicating a well-defined mesostructure with large accessible pores, and the specific surface area and pore volume are calculated to be 42.2 m^2^/g and 0.077 cm^3^/g, respectively. The pore size distributions calculated by the BJH method centered at 30.5 nm (Figure S11). The large surface areas of Si-WO_3_ HHSs could provide a large number of active sites for guest molecules (e.g., gas molecules) and would be favorable for promoting chemical reaction rates in heterogeneous catalysis and gas sensing.

X-ray photoelectron spectroscopy (XPS) characterizations of the Si-WO_3_ HHSs revealed core level peaks of W 4f, O 1s, and Si 2p (Figure 4d–f), confirming the presence of W, Si, and O species. The peak-differentiating and imitating analysis indicated the presence of a small amount of W^5+^ (22.5%), and the O 1s peak also revealed abundant (27.5%) surface-adsorbed oxygen species (O^–^, O_2_^–^), indicating rich oxygen vacancies in the crystal lattice of WO_3_. The above results confirmed that the Si atoms are successfully doped into a crystal lattice of tungsten oxide, thus causing the enhancement of surface-adsorbed oxygen species according to the charge compensation mechanism. Furthermore, the Si-doping enables the W atoms to deviate from the center of the W–O octahedron, resulting in an ε-phase WO_3_ with a high dipole moment. The Si content calculated from XPS (6.2 at %) is much higher than the theoretical content from the stoichiometric ratio of H_4_SiW_12_O_40_ (2.0 at %) (Figure 4f), indicating that little silicon-containing species (SiO_*x*_) were located on the surface of WO_3_ except for atomic doping into the crystal lattice.

Solid state ^29^Si MAS NMR spectra of Si-WO_3_ hollow hemispheres (Figure 4b) showed Q^1^, Q^2^, Q^3^, and Q^4^ peaks of the Si element. It indicates that except for the formation of amorphous SiO_2_ (Q^4^), there are abundant Si species embedded into the lattice of tungsten oxide with the form of Si^4+^ (Q^1^, Q^2^, Q^3^). Such a result coincides well with the XPS characterizations. The UV–vis absorption spectra and the WCl_6_-derived mesoporous WO_3_ and the H_4_SiW-derived Si-WO_3_ HHSs also showed different absorption curves within the range of 250–800 nm (Figure 4c), and the corresponding extracted band gap (*E*_g_) of Si-WO_3_ (2.76 eV) is significantly higher than that of WO_3_ (2.50 eV). The relationship between carrier concentration (*n*_*i*_) and band gap (*E*_g_) follows the equation: *n*_*i*_ = (*N*_c_*N*_v_)∧(1/2) exp[−*E*_g_/(2*kT*)]. Therefore, the higher band gap can result in lower carrier concentration. As a typical n-type semiconductor, the carrier concentration of Si-WO_3_ is dominated by number of electrons. In semiconductor gas sensing tests, according to the definition of sensitivity (*R*_a_*/R*_g_), it could be predicted that the higher carrier concentration formed in air would lead to a smaller variation in the density of the charge carrier by the gas sensing reaction, and lower carrier concentration would be more advantageous for achieving a high gas sensing response. As a result, when exposed to reducing gases, the sensor based on Si-WO_3_ may show higher response because of the higher variation of the charge carrier concentrations.

### Synthesis and Characterization of the Si-WO_3_ Nanoparticles and Nanowires

**Figure 5:**
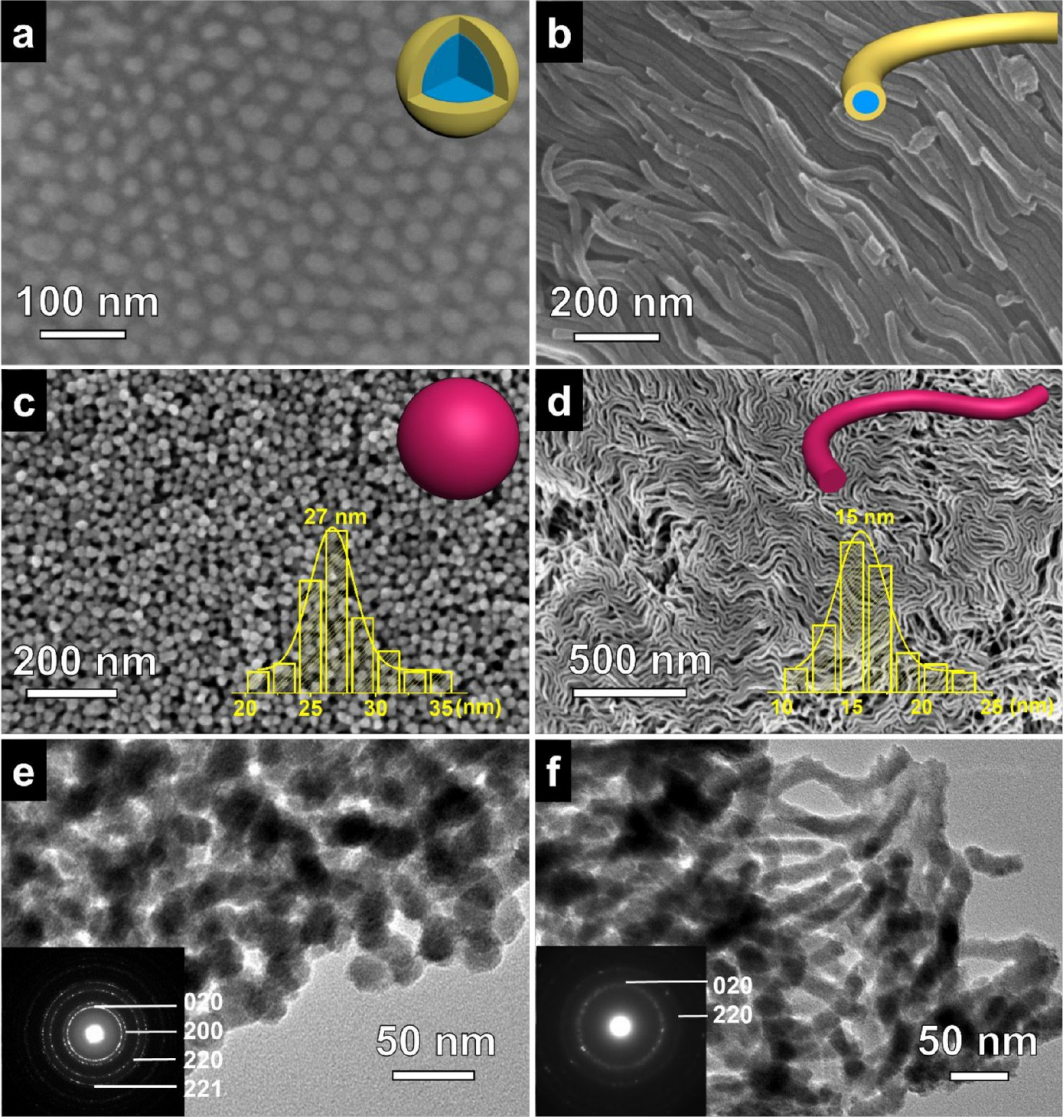
FESEM images of the PEO-*b*-PS/H_4_SiW_12_O_40_: (a) inverse spherical micelles, (b) inverse cylindrical micelles; FESEM and TEM images of the Si-WO_3_ (c, e) nanoparticles and (d, f) nanowires. The upper right insets in (a–d) are the corresponding structural models, and the insets in (e, f) are the corresponding selected area electron diffraction (SAED) patterns. The insets at the bottom of panels (c, d) are diameter distributions of the Si-WO_3_ (c) nanoparticles and (d) nanowires.

Following a similar casting process, nanocomposite films consist of PEO-*b*-PS/H_4_SiW_12_O_40_ inverse spherical micelles and inverse cylindrical micelles could also be constructed on the substrate (Figure 5a, b). After annealing at 500 °C in N_2_ and 400 °C in air, a film composed of Si-WO_3_ nanoparticles and nanowires were obtained (Figure S13–S16). As shown in Figure 5c, e, the Si-WO_3_ nanoparticles possessed uniform spherical morphology with a narrow size distribution of about 27 nm, and the Si-WO_3_ nanowires exhibited irregular-curved nanowires with a high aspect ratio (about 15 nm in diameter and several microns in length) (Figure 5d, f). In accordance with electron diffraction patterns from TEM, both Si-WO_3_ nanoparticles and nanowires are highly crystalline. Furthermore, the film composed of Si-WO_3_ NPs and NWs are highly porous due to the stacking of the NPs and NWs (Figure S17), endowing them potential for applications such as heterogeneous catalysis, gas sensing, and photoelectric conversions by virtue of their large amount of adsorption sites and semiconducting properties.

### Generality of the Coassembly Strategy

It is found that this dynamic coassembly in miscible *n*-hexane and THF mixed solvent can be used as a general and flexible approach to synthesize other nonmetal elements (including Si and P) doped transition metal oxides (WO_3_/MoO_3_) nanomaterials (Figure S18–S20). For example, following similar coassembly and the annealing process, P-WO_3_ nanobowls and nanoparticles, P-MoO_3_ nanobowls, and Si-MoO_3_ nanoparticles could be readily synthesized from the coassembly of PEO-*b*-PS with phosphotungstic acid (H_3_PW_12_O_40_), silicomolybdic acid (H_4_SiMo_12_O_40_), and phosphomolybdic acid (H_3_PMo_12_O_40_), respectively, in THF/*n-*hexane. Similar to synthesis of Si-WO_3_ nanomaterials, the hollow hemispheres and nanoparticles could be generated from constructing PEO-*b*-PS/POMs spherical vesicles and inverse spherical micelles, respectively. The Si-/P-doped metal oxides possessed uniform well-defined nanostructures, high specific surface areas, highly crystalline frameworks, and semiconducting properties (Figure S19, 20), and these doped metal oxide nanostructures hold great potential for applications in many applications such as heterogeneous catalysis, gas sensing and energy storage, and conversions.

### Gas Sensing Performances of the Si-WO_3_ Nanobowls

**Figure 6:**
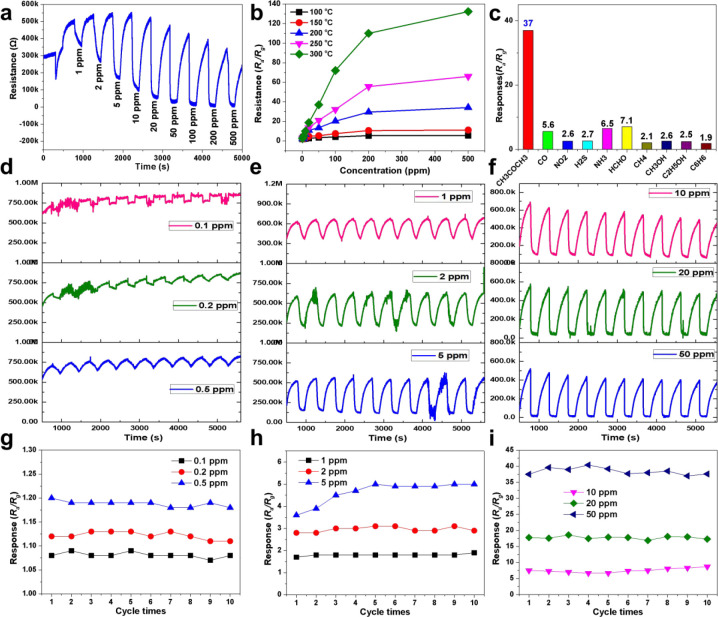
Gas sensing performances of Si-WO_3_ nanobowls-based sensor. (a) Response-recovery curves of the sensor to acetone of different concentrations (1–500 ppm) at 300 °C, (b) sensing responses of the Si-WO_3_ hollow hemispheres-based sensor to 1–500 ppm acetone at different temperatures, and (c) responses of the sensor to different gases of 50 ppm at 300 °C. Cycling tests to acetone with different concentrations: (d, g) 0.1–0.5 ppm, (e, h) 1–5 ppm, and (f, i) 10–50 ppm.

The gas sensing performances of the Si-WO_3_ HHSs materials were tested on a side-heated type gas sensor by a dynamic gas distribution test system (Figure 6, S21). First, the sensor was tested for 50 ppm acetone at different temperatures (100–350 °C) to obtain the optimum working temperature (Figure 6b, S22). With an increase of the working temperature, the sensing response (*S = R*_a_*/R*_g_) first rose and then went down, and the response time kept decreasing. The sensor reached its maximum response value (*S* = 37) at 300 °C, with a short response time (*t* = 7 s). Therefore, in this study, the optimal working temperature of the sensor was found to be around 300 °C. As shown in Figure 6a, the resistance signal drops rapidly when acetone gas was injected into the test chamber, and it can be completely restored to its initial value when the sensor was exposed to air. The response of the sensor rose from 2.0 to 132.3 as the acetone concentration increases gradually from 1 to 500 ppm (Figure 6b). Moreover, the sensor was tested to the subppm level acetone, and the sensitivity was 1.09, 1.13, and 1.19 to 0.1, 0.2, and 0.5 ppm acetone, respectively. Obviously, the limit of detection (LOD) is lower than 0.1 ppm. Furthermore, to evaluate its stability, the sensor was tested to 0.1–50 ppm acetone for ten cycles (Figure 6d–i), and all the response–recovery curves were repeated well, indicating a good long-term stability. Thanks to the porous nanostructures and crystalline framework that facilitates diffusion and transport of gases and electron conduction, respectively, the gas sensing device showed fast response-recovery dynamics. For example, in 50 ppm acetone atmosphere, the response time was as short as 7 s (Figure S23). The selectivity is also an important parameter for a gas sensor; in this study, nine volatile interference gases, including ethanol, hydrogen sulfide, benzene, formaldehyde, carbon monoxide, ammonia, methanol, nitrogen dioxide, and methane were introduced, respectively, to study the sensing response of the sensor. As shown in Figure 6c, the sensitivity of the sensor to 50 ppm acetone reaches 37, while all the interference gases show sensitivity of less than 7.2, at least five times lower than that in acetone. The specific selectivity toward acetone could be explained as follows. The Si-doping in lattice of WO_3_ formed a large amount of oxygen defects, and created abundant oxygen species adsorbed on the surface of WO_3_ in air, which facilitates the catalytic oxidation of reducing gas molecules at the working temperature. On the other hand, the Si-doping leads to lattice distortion of WO_3_. It can cause the W atoms to deviate from the center of the W–O octahedrons, leading to a spontaneous polarization effect and enhancing its adsorption capacity for gas molecules with a high dipole moment (*e.g*., acetone: 2.88 D). The increased gas–solid interface adsorption favors the catalytic oxidation of acetone molecules, resulting in a high sensing response.^40−43^ The high sensitivity toward acetone of the Si-WO_3_ sensor is very meaningful for applications of early diagnosis of diseases, as the acetone content in exhaled gas of type-I diabetes mellitus patients was significantly higher (>1.8 ppm) than that of healthy people.^41^

In order to clarify the effect of silicon doping on the gas sensing performance, the nondoped WO_3_ nanostructure was synthesized by using WCl_6_ as the tungsten source instead of silicotungstic acid, to coassemble with PEO-*b*-PS templates (Figure S24), and mesoporous nondoped WO_3_ was obtained and used to fabricate sensing devices toward acetone. As shown in Figure S25, the sensing response was 1.4, 2.3, 3.9, 7, 11.6, and 17.1 to 1, 2, 5, 10, 20, and 50 ppm acetone, respectively, at 300 °C. Compared to the Si-doped WO_3_ nanobowls (*R*_a_/*R*_g_ = 37.0 @ 50 ppm), the mesoporous WO_3_ based sensor showed much lower sensitivity (*R*_a_/*R*_g_ = 17.1) to acetone. These results further confirm that the interface interaction between guest gas molecules and host sensing materials is important to the sensitivity of gas sensors.

For comparison, we also fabricated semiconductor gas sensors based on Si-WO_3_, P-WO_3_, Si-MoO_3_, and P-MoO_3_ nanoparticles and investigated their gas sensing performances. As shown in Figures S26–29, the Si-WO_3_ sensor showed better acetone sensing performances than P-WO_3_ nanoparticles. The sensing response (*S = R*_air_*/R*_gas_) of the Si-WO_3_ nanoparticles to 50 ppm of acetone is *S* = 9.5, much higher than that of P-WO_3_ (*S* = 4.2). The *in situ* doping of Si atoms in the lattice of WO_3_ leads to more oxygen vacancies and surface-adsorbed oxygen species (O^–^, O_2_^–^), meanwhile it can increase the dipole moment of WO_3_. Therefore, the sensitivity of the gas sensor to acetone with a large dipole moment (*D* = 2.88) is dramatically improved. For P-WO_3_, P species are unstable and P_2_O_5_ would sublimate at high temperatures (>360 °C), thus resulting in less lattice defects and lower dipole moment, and the acetone sensing performance is not as good as Si-doped WO_3_, and its gas sensing behavior (*e.g*., selectivity) is close to the undoped WO_3_. Similarly, the Si-MoO_3_ showed higher sensitivity than P-MoO_3_ to reducing gases such as acetone (*S*_Si-MoO3_ = 2.8 and *S*_P-MoO3_ = 1.3) and ethanol (*S*_Si-MoO3_ = 3.8 and *S*_P-MoO3_ = 1.6).

### Study on Gas Sensing Mechanism

To investigate the gas sensing mechanism, *in situ* Fourier transform infrared spectroscopy (*in situ* FTIR) was conducted to reveal the catalytic reaction pathways of acetone on the surface of Si-WO_3_ (Figure S30). The Si-WO_3_ sample and KBr were pressed together, heated to 300 °C from room temperature, then acetone gas was injected into the chamber, and the infrared spectrum was monitored in real time. From the results we can observe that with an increase of the working temperature, the absorption peaks around 1737 cm^–1^ disappeared gradually, which was attributed to the carbonyl (C=O) in acetone, indicating that that acetone decomposes gradually. Meanwhile, new absorption peaks at 2306 and 2383 cm^–1^ appeared gradually, which could be referred to as carbon dioxide (CO_2_). No other peaks were observed during the testing process. From the above results, it could be concluded that the reaction pathways during the gas sensing process could be described as follows:
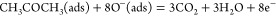
That is, acetone absorbed on the sensing materials was oxidized to carbon dioxide and water directly, and electrons flow from acetone to Si-WO_3_ materials, resulting in a resistance decrease of the sensing layer.

### Theoretical DFT Calculations

In order to further study the gas sensing mechanism in depth, a theoretical calculation based on density functional theory (DFT) was conducted to explain the high sensitivity and selectivity toward acetone of the Si-WO_3_ based sensor (Figure S31). The Si-WO_3_ structure was initially built and optimized, serving as a dominant interface region interacting with acetone molecules. The adsorption configurations of the (020) plane on the Si-WO_3_ is optimized, and the adsorption energies (*E*_ads_) for acetone was calculated to be −2.16 eV, indicating a strong adsorption capacity to acetone molecules of Si-WO_3_ (Figure S31a). In comparison, the optimized structures for the other nine interfering gases absorbed on Si-WO_3_ were also built, and the corresponding adsorption energies were calculated (Figure S32). Among all the investigated gases, *E*_ads_ for acetone was the most negative one (−2.16 eV). While for other gases, the adsorption energies are all higher than −1.0 eV (Figure S31i). It indicates that the (020) facets of Si-WO_3_ greatly benefit adsorption and subsequent catalysis of acetone compared with other gases. In addition, no obvious changes in molecular structures were observed after the adsorption of gas molecules onto the Si-WO_3_, and therefore, it can be proven that all the gases on the Si-WO_3_ are physically adsorbed. Meanwhile, the *E*_ads_ for acetone absorbed on the undoped WO_3_ was calculated to be −1.09 eV (Figure S31c), much higher than that of Si-WO_3_, indicating that Si doping is beneficial to the adsorption of acetone. The above results are consistent with the experimental results, further confirming the ultrahigh selectivity to acetone of the Si-WO_3_. Further calculations reveal that the band gap is narrowed after acetone absorption (Figure S31g, h), and the density of states (DOS) of the Si-WO_3_ + acetone displays a new energy level in the conduction band (Figure S31e, f), due to the strong bonding adsorption of acetone on the Si-WO_3_ and electron transfer from acetone to Si-WO_3_, which resulted in a decrease in resistance. These results consistently demonstrate that the acetone adsorption changes electronic structure and surface energy level of the Si-WO_3_.

Charge density difference was further calculated to elucidate the accurate electronic transfer during the acetone sensing process. As shown in Figure S31b, the blue and yellow lobes represent the charge depletion and accumulation, respectively, due to adsorption and oxidation of acetone molecules. The Bader charge analysis presents that there is 0.74 e of charge transfer (Δ*q*) from acetone to Si-WO_3_, while the Δ*q* is 0.56 e for acetone absorbed on undoped WO_3_ (Figure S31d), obviously lower than that of Si-WO_3_. This result is in accordance with the DOS results and charge distribution in the 2D plane of Si-WO_3_ (Figure S31k). In conclusion, the gas sensor based on the Si-WO_3_ nanobowls shows ultrahigh selectivity and sensitivity toward acetone because of the Si-WO_3_ that provides abundant active sites for acetone adsorption and accelerates electronic transfer during the gas sensing process.

### Construction of Gas Sensor Module

To further study the application possibility of the sensing devices, an advanced integrated gas sensor module based on the Si-WO_3_ sensing device was constructed for efficient real-time monitoring acetone concentration on a smart phone via Bluetooth communication. The module was composed of a Si-WO_3_ gas senor, a battery, a microcontroller unit, and a wireless data communication. As shown in Figure S33, the integrated sensor module was tested to 5, 10, 20, and 30 ppm acetone, respectively, and the sensor outputs a concentration signal of 4.5, 10.7, 20.5, and 28.8 ppm, respectively, with an error less than 10%. All the tests were repeated for three times, and the reproducibility was well maintained (Figure S34). The above results indicated that the sensor module is promising for application in real-time monitoring concentration of target gases.

## Conclusions

In summary, a dynamic coassembly of PEO-*b*-PS and H_4_SiW_12_O_40_ in THF/*n*-hexane dual solvent system has been developed to construct a variety of nanostructured Si-WO_3_ (nanobowls, nanoparticles, and nanowires). The THF/*n*-hexane volume ratio of the solution was found to dominate the morphologies of the assembled hybrid composites due to the changes of solubility of block copolymers and their assembly behaviors. The as-formed spherical vesicles, inverse spherical micelles, and inverse cylindrical micelles of PEO-*b*-PS/H_4_SiW_12_O_40_ were thermally converted into the above nanostructured Si-WO3 with distinct features. This synthetic strategy can be extended to the coassembly of PEO-*b*-PS with other Keggin-type POMs (H_3_PW_12_O_40_, H_4_SiMo_12_O_40_, and H_3_PMo_12_O_40_) to synthesize different kinds of heteroatom-doped transition metal oxides (P-WO_3_, Si-MoO_3_, and P-MoO_3_) with diverse nanostructures. The Si-WO_3_ hollow hemispheres-based semiconductor gas sensor exhibits excellent acetone sensing performances with high sensitivity and selectivity, due to its high specific surface areas and abundant surface adsorbed oxygen species originated from *in situ* Si doping. The excellent gas sensing performance makes it promising to develop miniaturized and wearable semiconductor gas sensors. Furthermore, this universal and flexible BCPs-POMs coassembly strategy offers great opportunity to design of various inorganic nanomaterials (metal carbide and metal nitride, *etc*.) with novel topological structures, abundant chemical compositions, and unique physicochemical properties, which show huge potentials in applications of catalysis, sensing, and energy storage and conversion.

## Experimental Section

### Chemicals

H_4_SiW_12_O_40_·15H_2_O, AR, Aladdin; H_3_PW_12_O_40_·21H_2_O, AR, Sigma-Aldrich; H_4_SiMo_12_O_40_·30H_2_O, AR, Aladdin; and H_3_PMo_12_O_40_·30H_2_O, AR, Aladdin. All chemicals were used without further purification.

### Synthesis of the Si-WO_3_ and P-WO_3_ Hollow Hemispheres, Nanoparticles, and Nanowires

In a typical synthesis, 0.05 g of the lab-made amphiphilic block copolymer PEO_114_-*b*-PS_156_ (*M*_n_ = 21500 g/mol, PDI = 1.06) synthesized according to our previous report^48^ was dissolved in 3 mL THF, to form the homogeneous solution A. 0.15 g of H_4_SiW_12_O_40_·15H_2_O (or H_3_PW_12_O_40_·21H_2_O) was dissolved in 1 mL THF to form the precursor solution B. The solution A and B were mixed to form a light blue transparent colloidal solution with further stirring for 0.5 h, then 3 mL *n*-hexane was added dropwise to form a white colloidal solution, which was then poured into a Petri dish to evaporate the solvent at room temperature for 12 h, followed by sequential heating at 100 °C for 24 h. Finally, the as-cast film was calcined at 500 °C for 1 h in N_2_ atmosphere (heating rate: 1 °C/min from room temperature to 350 and 5 °C/min from 350 to 500 °C) and 400 °C for 30 min in air (heating rate: 5 °C/min), and the sample of Si-WO_3_ (or P-WO_3_) hollow hemispheres was obtained.

The synthetic process for Si-WO_3_ (or P-WO_3_) nanoparticles and nanowires was the same with that of the Si-WO_3_ (or P-WO_3_) hollow hemispheres, except that the volume of *n*-hexane was 4 and 5 mL, respectively, and for nanowires the mass ratio of BCPs/POMs is 1/4.

### Synthesis of the Si-MoO_3_ and P-MoO_3_ Hollow Hemispheres and Nanoparticles

In a typical synthesis, 0.05 g of the lab-made amphiphilic block copolymer PEO_114_-*b*-PS_156_ (*M*_n_ = 21500 g/mol, PDI = 1.06) was dissolved in 3 mL THF to form the homogeneous solution A. 0.10 g of H_4_SiMo_12_O_40_·30H_2_O (or H_3_PMo_12_O_40_·30H_2_O) was dissolved in 1 mL THF to form the precursor solution B. The solution A, B and *n*-hexane (3 mL for hollow hemispheres, 4 mL for nanoparticles) were mixed to form a transparent colloidal solution with further stirring for 0.5 h, which was then poured into a Petri dish to evaporate the solvent at room temperature for 12 h, followed by sequential heating at 100 °C for 24 h. Finally, the as-cast film was calcined at 350 °C for 3 h in N_2_ atmosphere (heating rate: 1 °C/min) and at 350 °C for 2 h in air (5 °C/min), and the sample of Si-MoO_3_ (or P-MoO_3_) hollow hemispheres (or nanoparticles) was obtained.

### Synthesis of mesoporous WO_3_

0.1 g of PEO_114_-*b*-PS_156_ was dissolved in 5 mL THF, forming homogeneous solution A. 0.3 g of WCl_6_ was dissolved in 1 mL EtOH and 0.5 mL acetylacetone to form the precursor solution B. The solution A, B was mixed to form a green transparent solution with further stirring for 0.5 h, which was then poured into a Petri dish to evaporate the solvent at room temperature for 12 h, followed by sequential heating at 100 °C for 24 h. Finally, the as-cast film was calcined at 500 °C for 1 h in N_2_ atmosphere (heating rate: 1 °C/min below 350 and 5 °C/min above 350 °C) and 400 °C for 1 h in air (heating rate: 5 °C/min), and mesoporous WO_3_ was obtained.

### *In Situ* X-ray Photoelectron Spectroscopy Measurements

X-ray photoelectron spectroscopy measurements were carried out with an AXIS Supra by Kratos Analytical Inc. using monochromatized Al Ka radiation (*hv* = 1486.6 eV, 225 W) as an X-ray source with a base pressure of 10^–9^ Torr. Survey scan spectra were acquired using a pass energy of 160 eV and a 1 eV step size. Narrow region scans were acquired using a pass energy of 40 eV and a 0.1 eV step size. The hybrid lens mode was used in both cases. The analyzed area of all XPS spectra was 300 × 700 μm^2^. A charge neutralizer was used throughout as the samples were mounted such that they were electrically isolated from the sample bar. All spectra were calibrated by C 1s (284.8 eV). This surface was sputtered using the Ar-GCIS beam (*n* = 1000, 10 keV beam energy); the sputtering area is 3 × 3 mm^2^. The etch rate was ∼30 nm/min for poly(lactic-co-glycolic acid (PLGA, standard).

## Supporting Information Available

Gas sensing and computational methods; schematic diagram of synthesis mechanism (Figure S1); structural characterizations (Figure S2–S20); experimental details of gas sensing (Figure S21–29); SEM/TEM characterizations and gas sensing performances of mesoporous WO_3_ (Figure S24, S25); *in situ* Fourier transform infrared spectroscopy (Figure S30); DFT calculations (Figure S31, S32); and tests of real-time monitoring via Bluetooth communication (Figure S33, S34) (PDF)
