# Supplementary material for: Dynamic Coassembly of Amphiphilic Block Copolymer and Polyoxometalates in Dual Solvent Systems: An Efficient Approach to Heteroatom-Doped Semiconductor Metal Oxides with Controllable Nanostructures
Source: ACS Cent Sci. 2022 Jul 26;8(8):1196–208. doi: 10.1021/acscentsci.2c00784 (PMC9413427; doi:10.1021/acscentsci.2c00784)
Supplement: Supplementary file 1 — oc2c00784_si_001.pdf [file oc2c00784_si_001.pdf]

Supporting Information

Dynamic Coassembly of Amphiphilic Block Copolymer and Polyoxometalates in Dual Solvent Systems: An Efficient Approach to Heteroatom-Doped Semiconductor Metal Oxides with Controllable Nanostructures

Yuan Ren[†], *Wenhe Xie*[†], *Yanyan Li*[†], *Yuanyuan Cui*[‡], *Chao Zeng*[&], *Kaiping Yuan*[§*], *Limin Wu*[⊥*], *Yonghui Deng*[†⊥*]

[†]Department of Chemistry, Department of Gastroenterology, Zhongshan Hospital of Fudan University, State Key Laboratory of Molecular Engineering of Polymers, Shanghai Key Laboratory of Molecular Catalysis and Innovative Materials, Fudan University, Shanghai 200433, P. R. China.

[‡]Shimazu China Co LTD, Shanghai 200233, P. R. China

[&]School of Microelectronics, Fudan University, Shanghai 200433, P. R. China.

[§]Frontier Institute of Chip and System, State Key Laboratory of ASIC and System, Fudan University, Shanghai 200433, P. R. China.

[⊥]Institute of Energy and Materials Chemistry, Inner Mongolia University, 235 West University Street, Hohhot, 010021, China

*Email: kpyuan@fudan.edu.cn, wlm@imu.edu.cn, yhdeng@fudan.edu.cn

Table of Content

Supplementary gas sensing and computational methods (Page S2−S5)

Supplementary schematic diagram of synthesis mechanism (Page S6)

Supplementary structural characterizations (Page S6−S18)

Supplementary experimental details of gas sensing (Page S18−19)

Characterizations and gas sensing performances of mesoporous WO_3 (Page S20)

Gas sensing performances of P-WO_3, Si-MoO_3 and P-MoO_3 nanomaterials-based sensor (Page S21−23)

In situ FTIR spectroscopy (Page S24)

DFT calculations (Page S24−25)

Tests of real-time monitoring of gas concentration on smart phone (Page S26−27)

Supplementary Experiments

1. Characterizations and measurements.

Field emission scanning electron microscopy (FESEM) was performed on a Zeiss Ultra 55 field-emission SEM (Germany) operated at 3 kV and 10 μA. Transmission electron microscopy (TEM) was conducted on a JEM-2100 F microscope (JEOL, Japan) operated at 200 kV. The samples for TEM measurements were first dispersed in ethanol and supported onto a carbon coated copper grid. Nitrogen sorption isotherms were measured at 77 K with a Micromeritics Tristar 3020 analyzer. Before measurements, the samples were degassed in vacuum at 180 °C for at least 6 h. The specific surface area and the pore size distribution were calculated by using the Brunauer-Emmett-Teller (BET) and Barrett-Joyner-Halenda (BJH) method, respectively. The total pore volume (V_{total}) was calculated from the adsorption volume at P/P_0 = 0.995. Fourier-transform infrared (FTIR) spectra were collected on a Nicolet Fourier spectrophotometer using KBr pellet method. Powder X-ray diffraction (XRD) patterns were recorded on Bruker D4 X-ray diffractometer (Germany) equipped with Ni-filtered Cu Kα radiation (40 kV, 40 mA). The X-ray photoelectron spectroscopy (XPS) spectra were collected on an RBD 147 upgraded PHI 5000C ESCA system with a dual X-ray source. The Mg Kα (1253.6 eV) anode and a hemispherical energy analyzer were used in the measurements. All of the binding energies were referenced to the C 1s peak at 284.8 eV of the surface adventitious carbon. The average sizes of micelles were tested using a dynamic light scattering (DLS) instrument (Malven, Zetasizer Nano ZS, UK).

2. Gas sensing tests

Before the tests, the Si-WO_3 hollow hemispheres powder was mixed with ethanol and grounded in an agate mortar to form a paste. The resulting paste was coated on an alumina tube on which a pair of Au electrodes had been printed previously, followed by drying at 100 °C for about 2 h and subsequently annealing at 250 °C for about 2 h. Finally, a small Ni-Cr alloy coil was inserted into the tube as a heater to adjust and optimize the working temperature of the gas sensor. To improve the long-term stability, the sensors were kept at the working temperature for 3 days. The gas sensing performance of the fabricated Si-WO_3

sensors was performed using a dynamic gas distribution test system (JF02F, China). The gas sensing response (*S*) in the measurement was deduced using the following equation, $S=R_a/R_g$, where R_a and R_g are the resistance of materials in air and in the test gas, respectively. In addition, the gas sensing performance working at different temperatures was investigated to find the optical working temperature, which is determined by the voltage of the heating electrode. The commercial acetone gas was purchased from Shanghai Weichuang Company. The acetone in the dry air with an accurate concentration can be controlled by the gas distribution box.

3. Computational methods

First-principles were used to describe the ions behavior in the anode based on density functional theory (DFT) with the Vienna ab initio simulation package (VASP) code. In addition, Perdewe-Burkee-Ernzerhof (PBE) generalized gradient approximation and the projected augmented wave (PAW) method were used to describe the ion-electron interactions in our systems. In this study, the plane-wave cutoff energy was set to 450 eV, and van der Waals corrections (optPBE-vdW) were adopted during structural optimization for the layer materials, and the vdWs interactions were described exactly by using DFT-D3 correction method of Grimme's scheme. Partial occupancies of the Kohn−Sham orbitals were allowed using the Gaussian smearing method and a width of 0.05 eV. The electronic energy was considered self-consistent when the energy change was smaller than 10^{-6} eV. A geometry optimization was considered convergent when the energy change was smaller than 0.05 eV $Å^{-1}$. The vacuum spacing in a direction perpendicular to the plane of the structure is 15 Å. The Brillouin zone integration is performed using 3×3×1 Monkhorst-Pack k-point sampling for a structure. Finally, the adsorption energies (E_{ads}) were calculated as $E_{ads}= E_{ad/sub} -E_{ad} -E_{sub}$, where $E_{ad/sub}$, E_{ad}, and E_{sub} are the total energies of the optimized adsorbate/substrate system, the adsorbate in the structure, and the clean substrate, respectively.

4. Principle of wireless gas sensor module

The circuit diagram of the wireless gas sensor module is as follows:

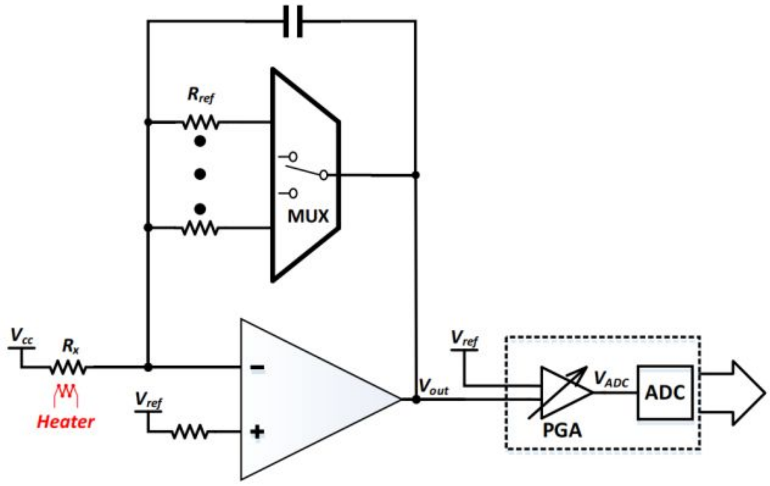

The inverting amplifier can convert the resistance information into voltage information. The operational amplifier OPA376 (Texas Instrument, TI) was adopted in the system, which provides extremely high input impedance and rail to rail input/output range.

MUX (multiplexer) is connected to the feedback resistance of the inverting amplifier as a switch, which can change the measuring range of the resistance. In this system, ADG704 from Analog Devices Company is adopt as MUX to provide extremely low on-resistance (<8 Ω) to ensure measurement accuracy, and extremely low leakage current (<0.3 nA) to ensure large resistance measurement in the order of GΩ.

ADS1115 from TI Company was adopted as the Analog to Digital Converter (ADC). The ADC was integrated with a differential input Programmable Gain Amplifier (PGA), which can be used to realize voltage subtraction calculation. The voltage is collected by ADC and processed by Micro Controller Unit (MCU). The resistance value can be calculated and the concentration can be fitted, which can be sent to the smart phone via Bluetooth.

In addition, the heating power of the heating resistor can be controlled by Pulse Width Modulation (PWM), and the heating current can be collected by measuring the voltage of the resistor in series, and the data can be sent to the MCU for power management or sent to a smart phone.

According to the schematic,

$$\frac{V_{ref1}-V_{cc}}{R_x} = \frac{V_{out}-V_{ref1}}{R_{ref}}$$

$$V_{out} = V_{ref1} + \frac{R_{ref}}{R_x} \cdot \left(V_{ref1} - V_{cc}\right)$$

in which, V_{out} is the output voltage, V_{ref1} is the voltage reference, R_x is the resistance value of gas sensor, and V_{cc} is the supply voltage.

The voltage collected by ADC is

$$V_{ADC} = A_{PGA} \cdot \left(V_{ref2} - V_{out}\right) = A_{PGA} \cdot \frac{R_{ref}}{R_x} \cdot \left(V_{cc} - V_{ref2}\right)$$

where A_{PGA} is the voltage gain of PGA inside ADS1115.

When the resistance to be measured is too small, the voltage measured by the ADC is relatively large. The measurement range of the circuit can be adjusted by reducing the PGA gain A_{PGA}, or switching the reference resistance through MUX (reducing the value of R_{ref}).

When the resistance to be measured is too large, the voltage measured by the ADC is low. The measurement range of the circuit can be adjusted by increasing the PGA gain A_{PGA}, or switching the reference resistance through MUX (increase the value of R_{ref}).

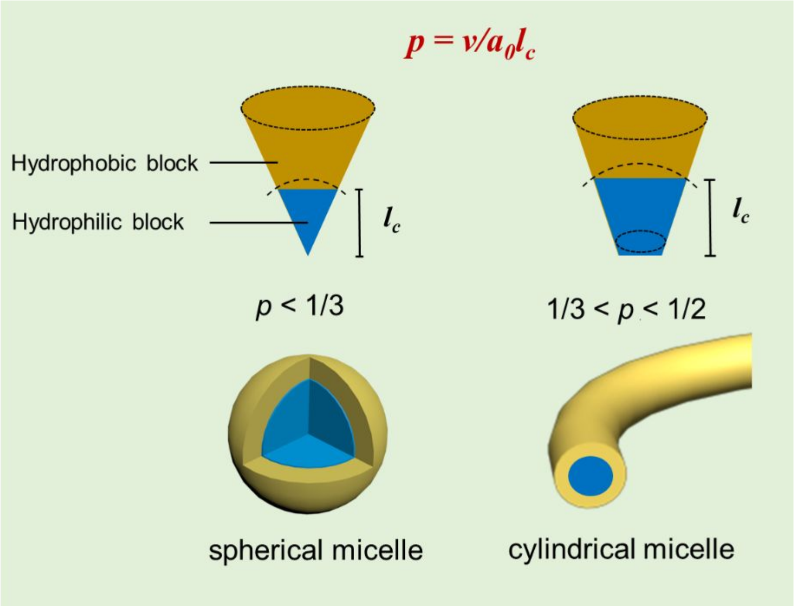

Figure S1. Schematic illustration of the micelle structures formed by the coassembly of block copolymer and polyoxometalates in *n*-hexane/THF dual solvent solution. The self-assembled structures are determined by the packing parameter (p).

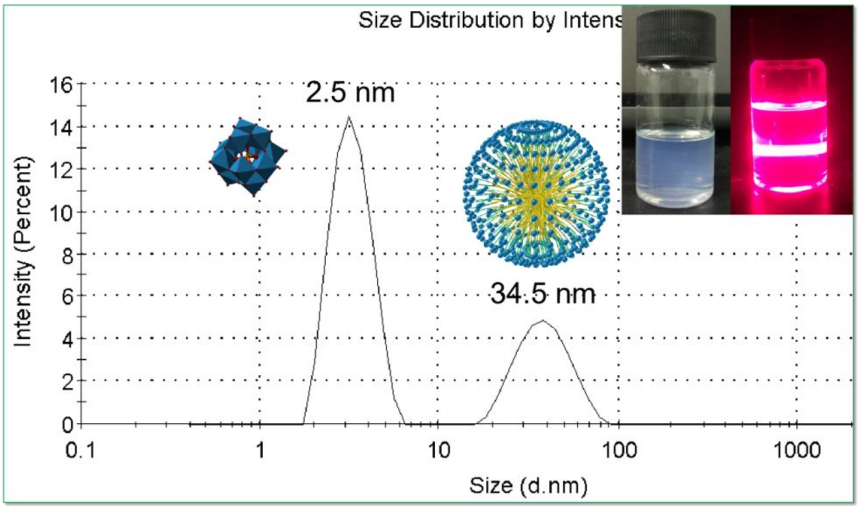

Figure S2. Dynamic light scattering (DLS) characterization of the PEO-*b*-PS/H_4SiW/THF colloidal solution, showing an interesting bimodal narrow size distribution. The first distribution peak centered at 34.5 nm corresponds to PEO-*b*-PS/H_4SiW spherical micelles, and the peak at 2.5 nm is attributed to free $SiW_{12}O_{40}^{4-}$ anions. The insets are optical photograph and the Ttindal effect of the PEO-*b*-PS/H_4SiW/THF colloidal solution.

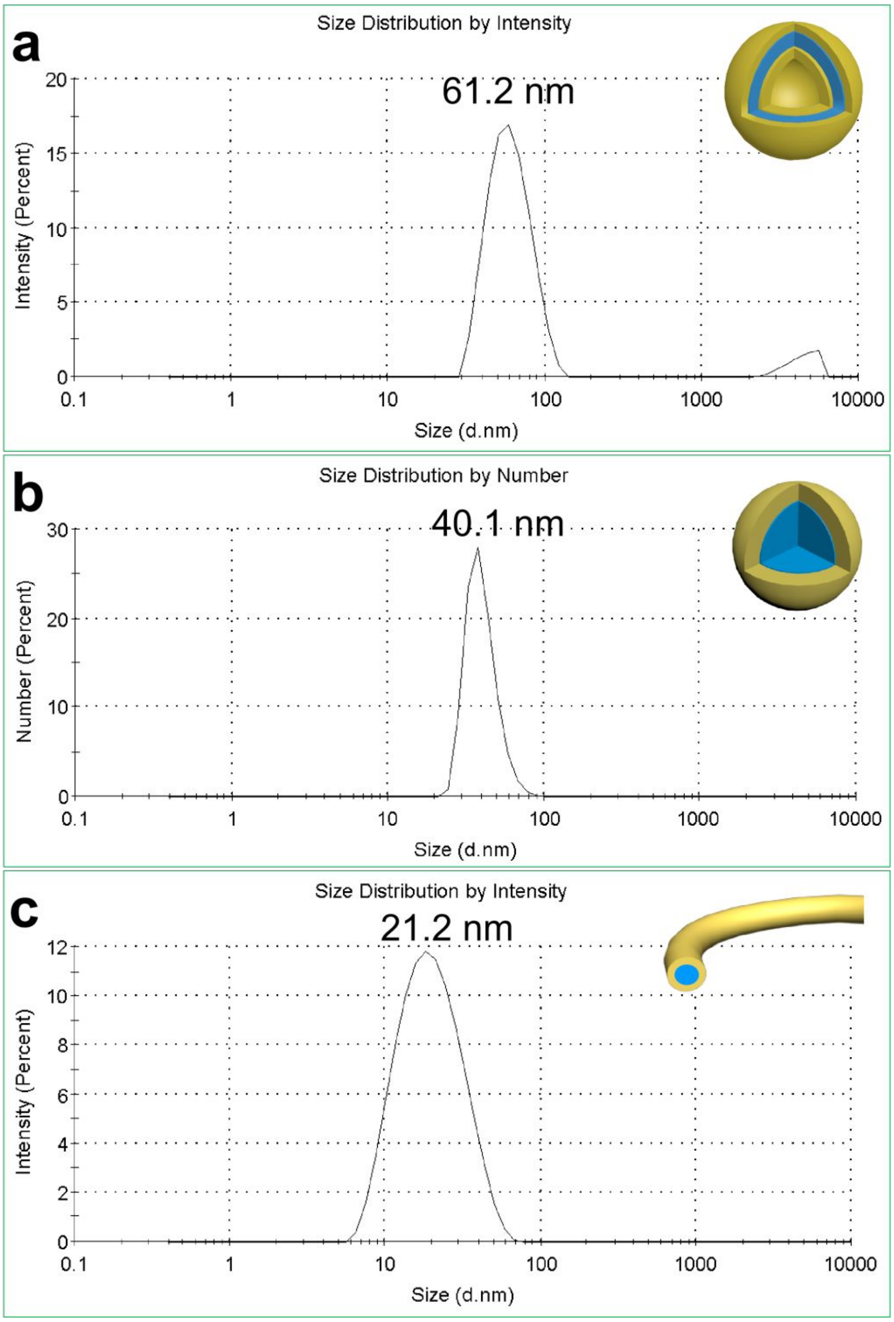

Figure S3. Dynamic light scattering (DLS) characterization of the PEO-*b*-PS/H_4SiW/THF/*n*-hexane colloidal solution containing (a) spherical vesicles, (b) inverse spherical micelles and (c) inverse cylindrical micelles.

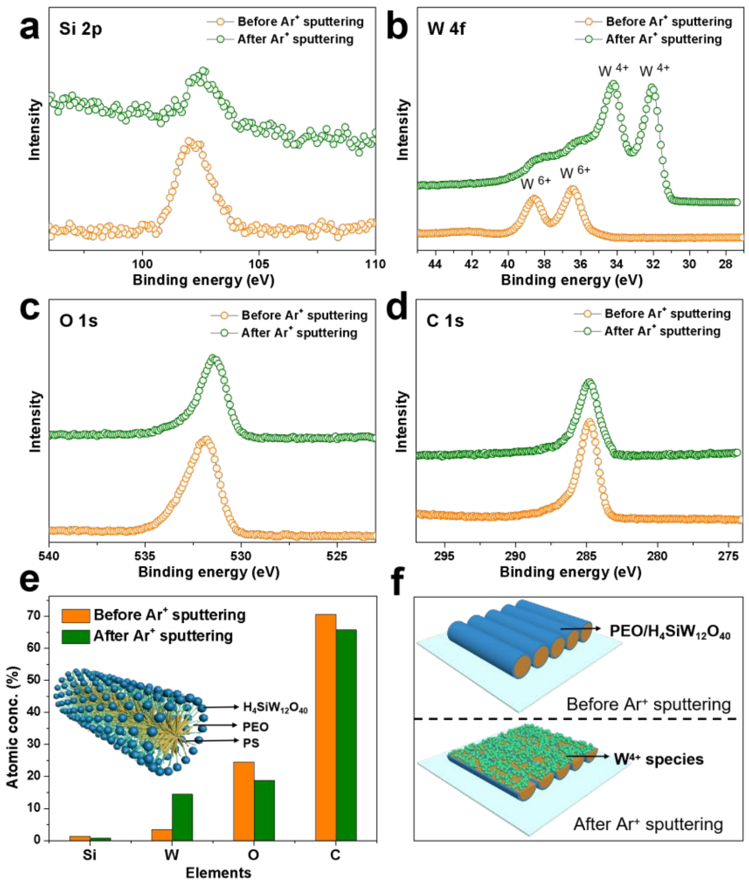

Figure S4. X-ray photoelectron spectroscopy (XPS) of PEO-*b*-PS/$H_4SiW_{12}O_{40}$ cylindrical micelles (without *n*-hexane) supported on silicon wafer before and after Ar^+ sputtering: (a) Si 2p, (b) W 4f, (c) O 1s and (d) C 1s. (e) Element contents measured by XPS and (f) the corresponding structural models of the PEO-*b*-PS/$H_4SiW_{12}O_{40}$ cylindrical micelles before and after Ar^+ sputtering.

Firstly, the nanocomposites film composed of PEO-*b*-PS/$H_4SiW_{12}O_{40}$ cylindrical micelles was tested without treatment, the elements content calculated by XPS results was Si = 1.38 at%, W = 3.46 at%, O = 24.51 at% and C = 70.65 at% respectively. After Ar^+ sputtering for 180 s, the Si and O content was decreased to 0.82 at% and 14.53 at% respectively, which was caused by etching of PEO/$SiW_{12}O_{40}{}^{4-}$ species on the surface of the film, conforming the PEO-*b*-PS/$H_4SiW_{12}O_{40}$ cylindrical micelles structure with PS chain as core and PEO/$SiW_{12}O_{40}{}^{4-}$ domain as shell. The unpredictable rise of W content could be explained by that it is difficult to be etched, the sputtering speed is much slower compared with the surrounding Si, C, O elements, which agrees well with the appearance of W species with low chemical state (W^{4+}).

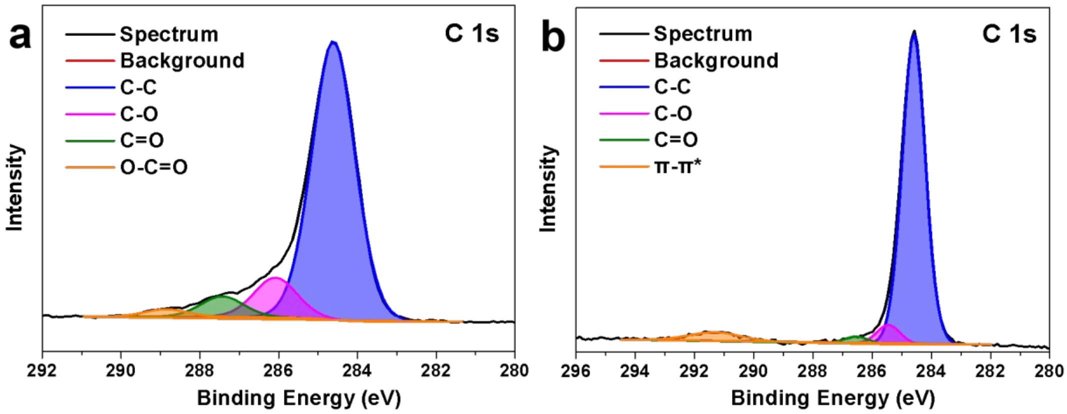

Figure S5. X-ray photoelectron spectroscopy (XPS) showing the C 1s core level peak regions of PEO-*b*-PS/$H_4SiW_{12}O_{40}$ hybrid film supported on silicon wafer before Ar^+ sputtering: (a) cylindrical micelles, (b) spherical vesicles.

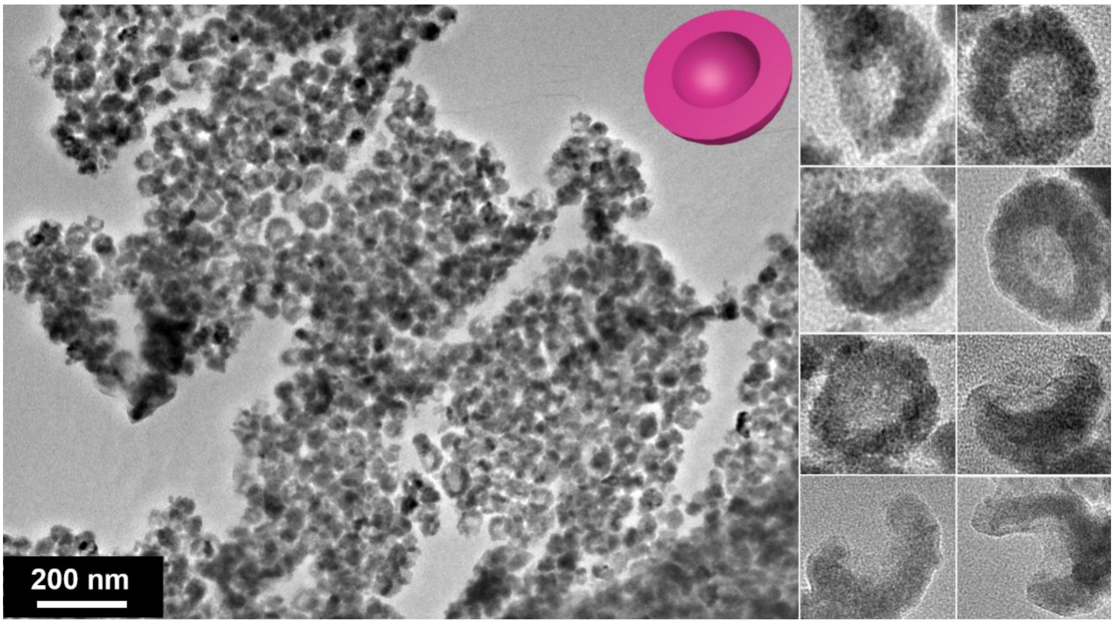

Figure S6. TEM images at low magnification and from different directions at high magnification of the Si-WO_3 hollow hemispheres, showing a nanobowl-like morphology.

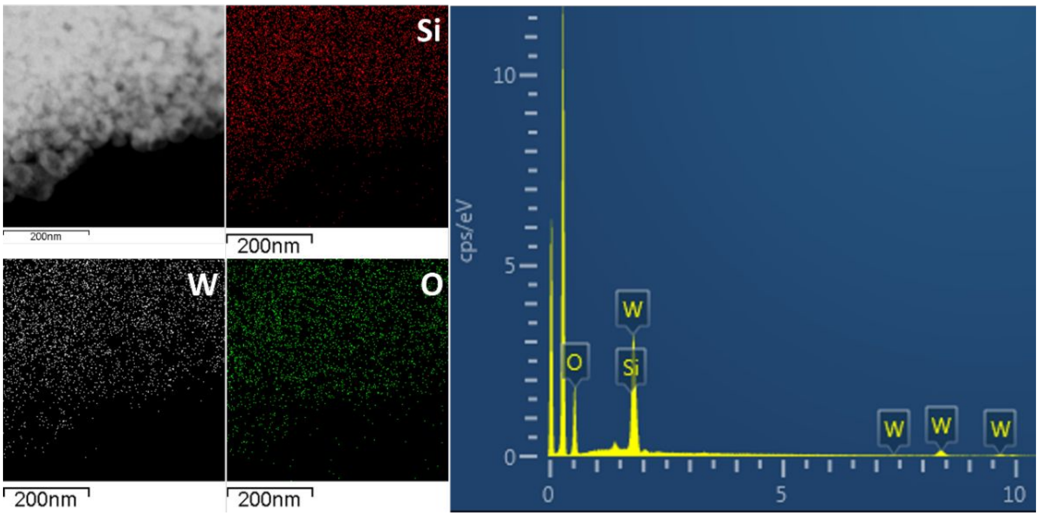

Figure S7. Elemental mapping (Si, O, W) and EDS pattern of the Si-WO_3 nanobowls.

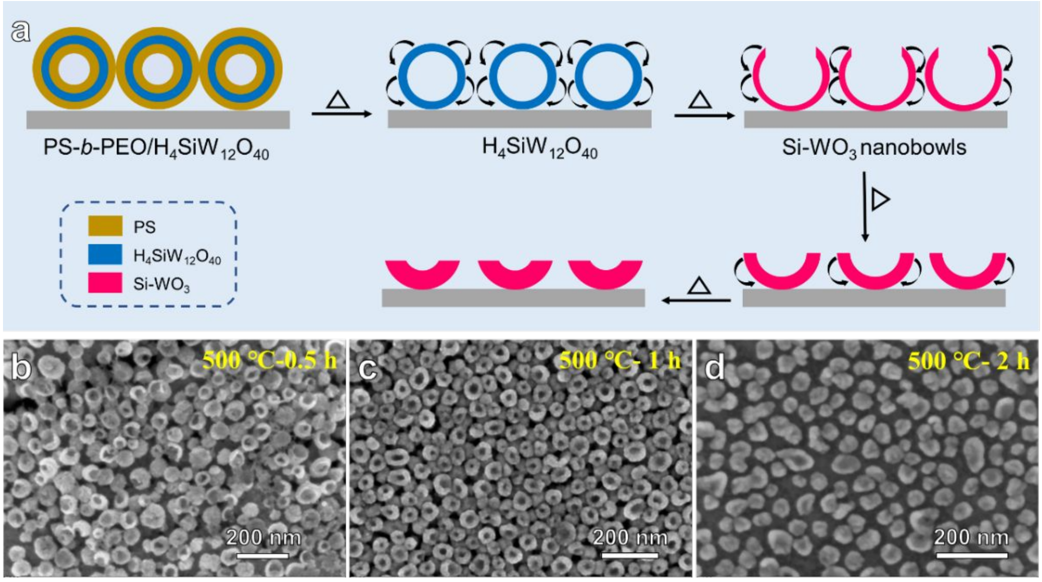

Figure S8. (a) Schematic illustration of the structural evolution of PEO-*b*-PS/$H_4SiW_{12}O_{40}$ spherical vesicles into Si-WO_3 nanobowls via a thermal-induced structural transformation process, SEM image of the Si-WO_3 nanobowls obtained after calcination at 500 ℃ for different time: (b) 0.5 h, (c) 1.0 h, (d) 2.0 h.

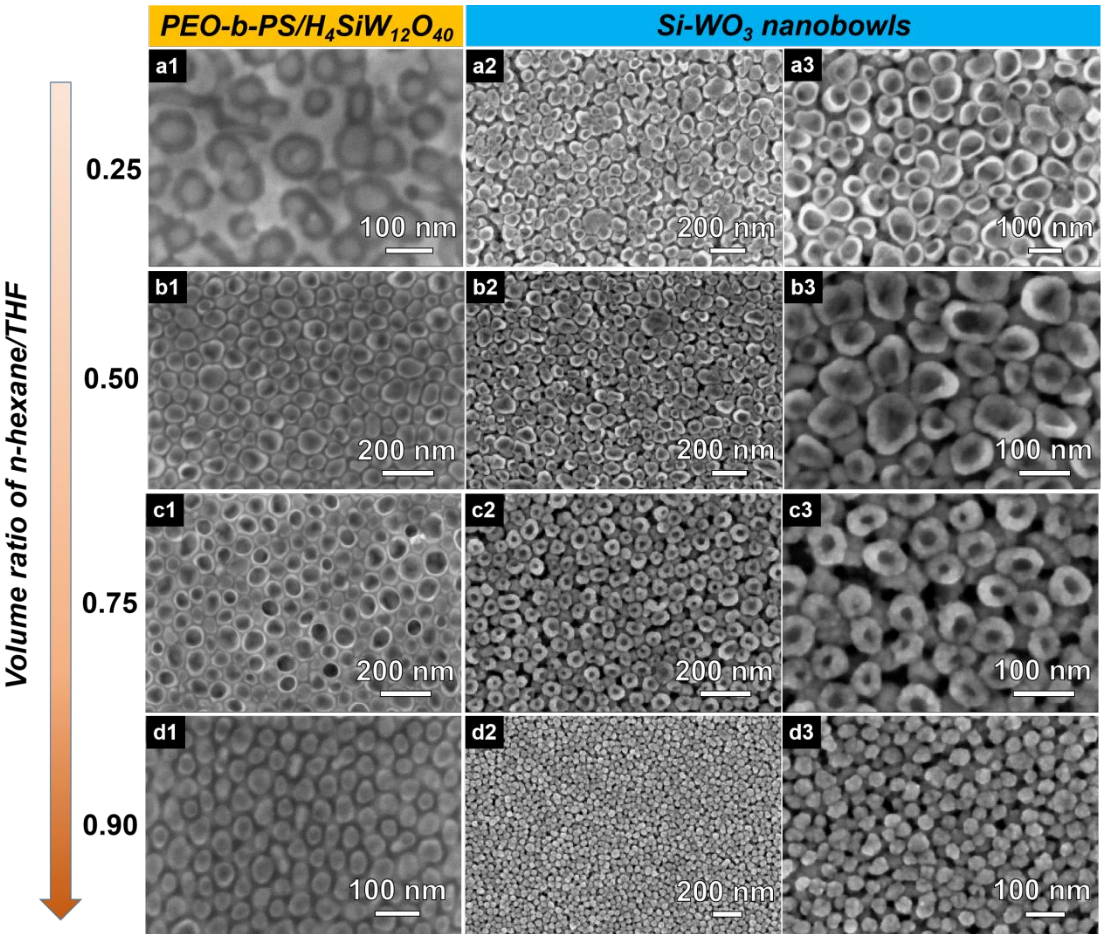

Figure S9. FESEM images of the PEO-*b*-PS/$H_4SiW_{12}O_{40}$ nanocomposite films and Si-WO_3 nanobowls generated from synthesis with different volume ratio of *n*-hexane/THF: (a) 0.25, (b) 0.50, (c) 0.75, (d) 0.90.

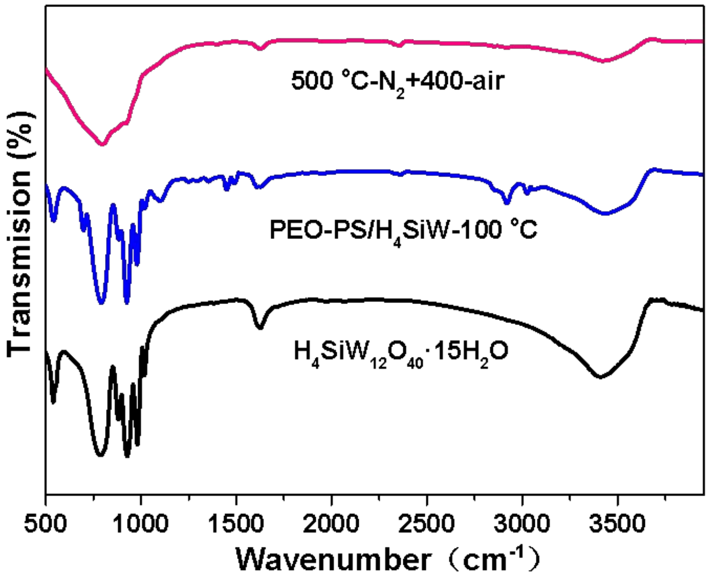

Figure S10. FTIR spectrums of the commercial $H_4SiW_{12}O_{40}·15H_2O$, PEO-*b*-PS/$H_4SiW_{12}O_{40}$ nanocomposites treated at 100 °C, and Si-WO_3 hollow hemispheres obtained after thermal treatment at 500 °C in N_2 and 400 °C in air.

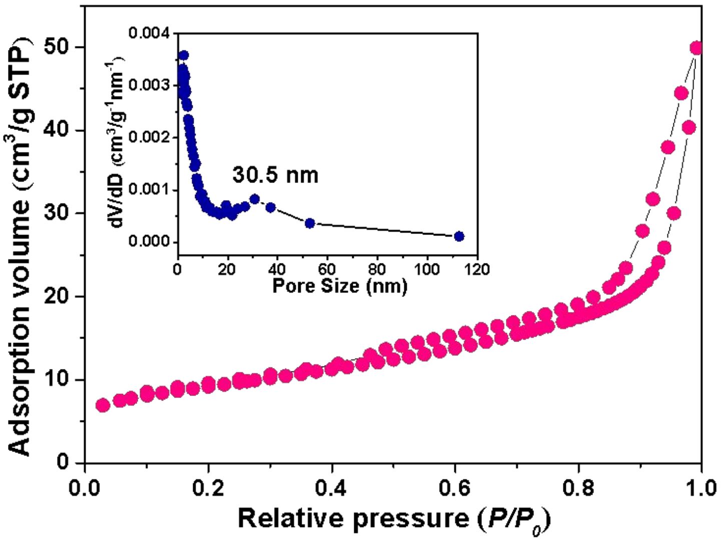

Figure S11. Nitrogen adsorption-desorption isotherms and the corresponding pore size distribution curve of Si-WO_3 hollow hemispheres.

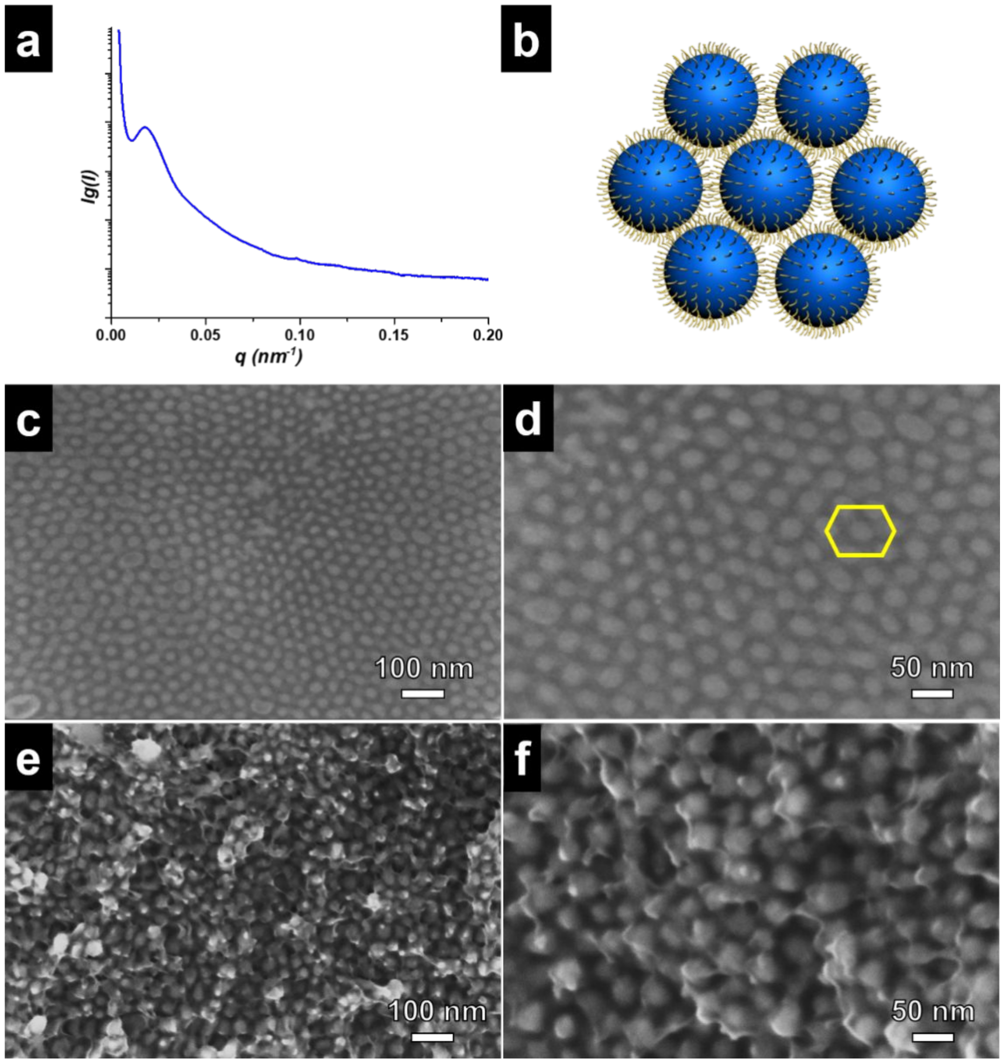

Figure S12. (a) SAXS pattern, (b) structural model, SEM images from (c, d) top view and (e, f) cross-section of the hybrid film composed of the H_4SiW/PEO-*b*-PS inverse spherical micelles.

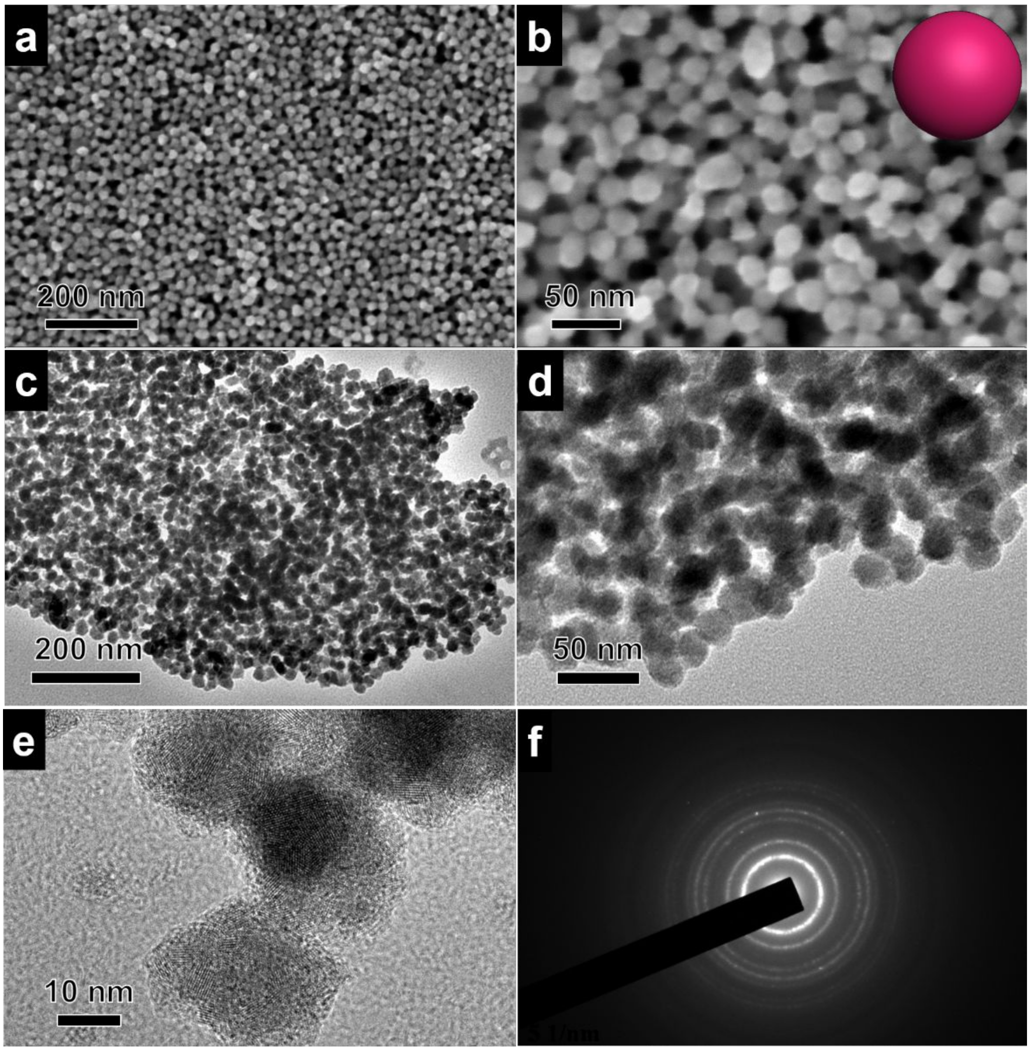

Figure S13. (a, b) FESEM, (c, d) TEM, (e) HRTEM images and (f) SAED pattern of the Si-WO_3 nanoparticles.

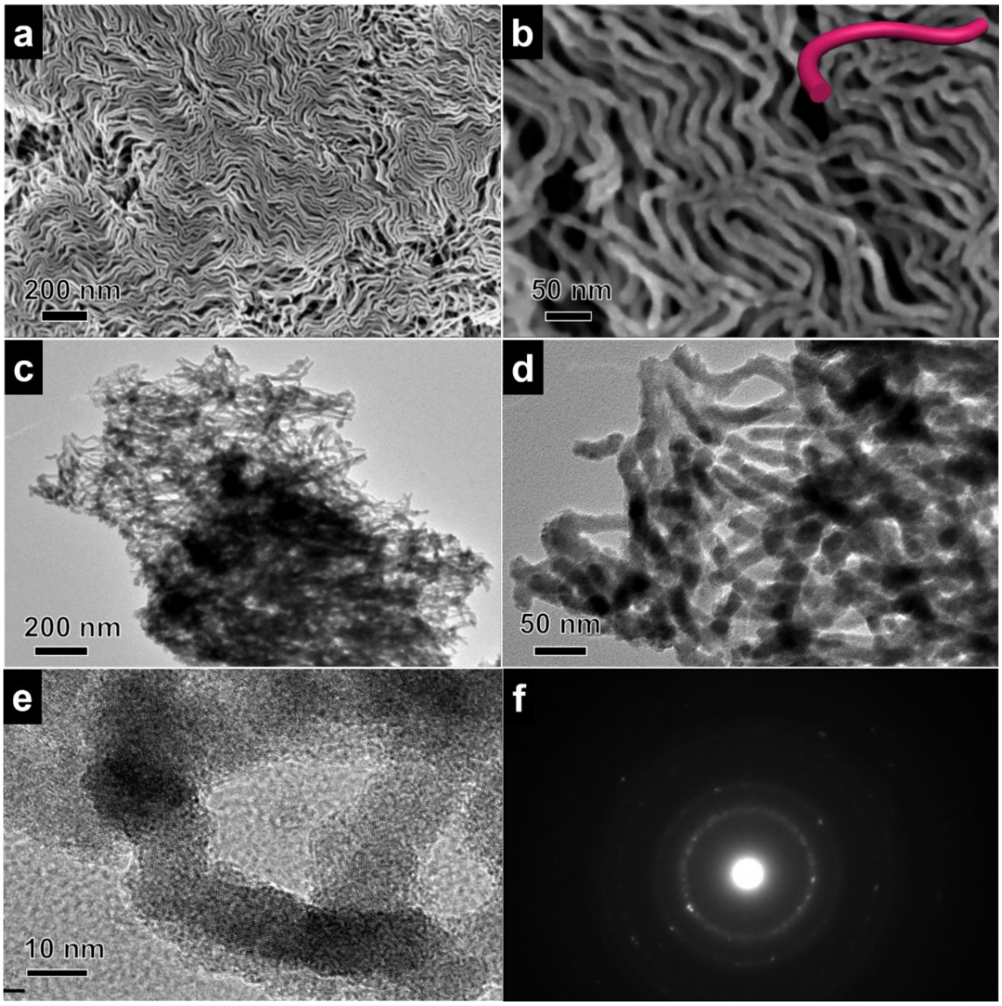

Figure S14. (a, b) FESEM, (c, d) TEM, (e) HRTEM images and (f) SAED pattern of the $Si\text{-}WO_3$ nanowires.

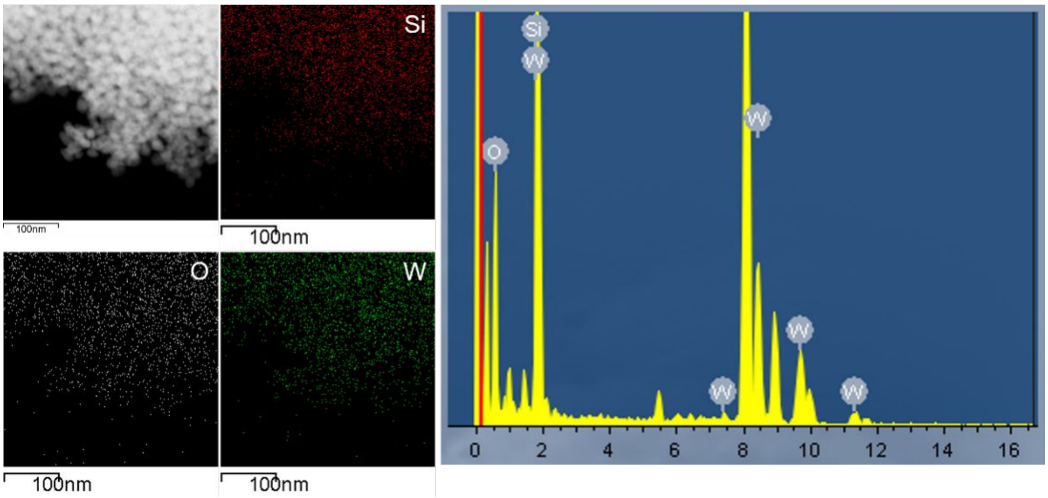

Figure S15. Elemental mapping (Si, O, W) and EDS pattern of the $Si\text{-}WO_3$ nanoparticles.

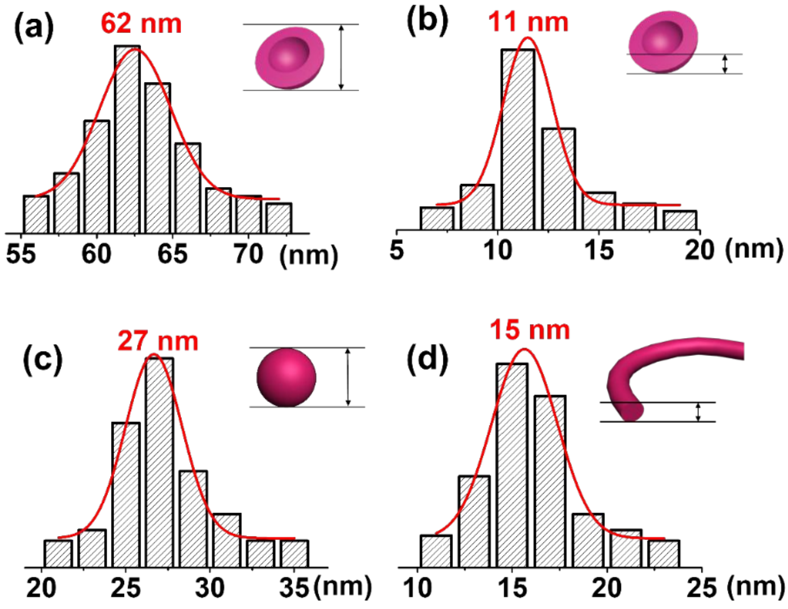

Figure S16. (a) Diameter and (b) thickness distribution of the Si-WO_3 nanobowls in **Figure 3d**, diameter distribution of the Si-WO_3 (c) nanoparticles and (d) nanowires in **Figure 5c, d**.

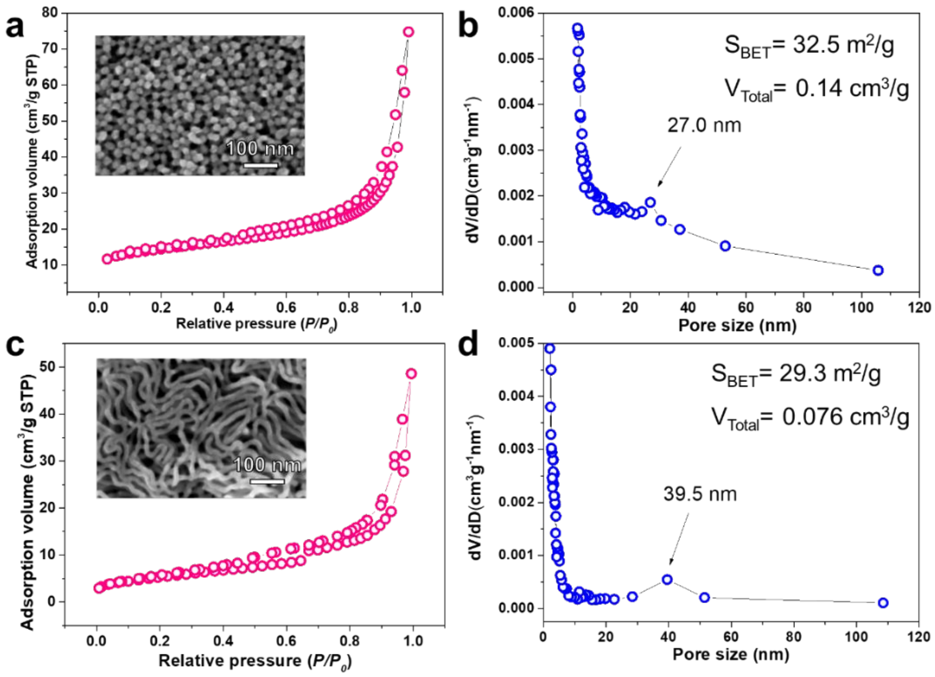

Figure S17. (a, c) Nitrogen adsorption-desorption isotherms and (b, d) the corresponding pore-size distribution curves of the Si-WO_3 (a, b) nanoparticles and (c, d) nanowires.

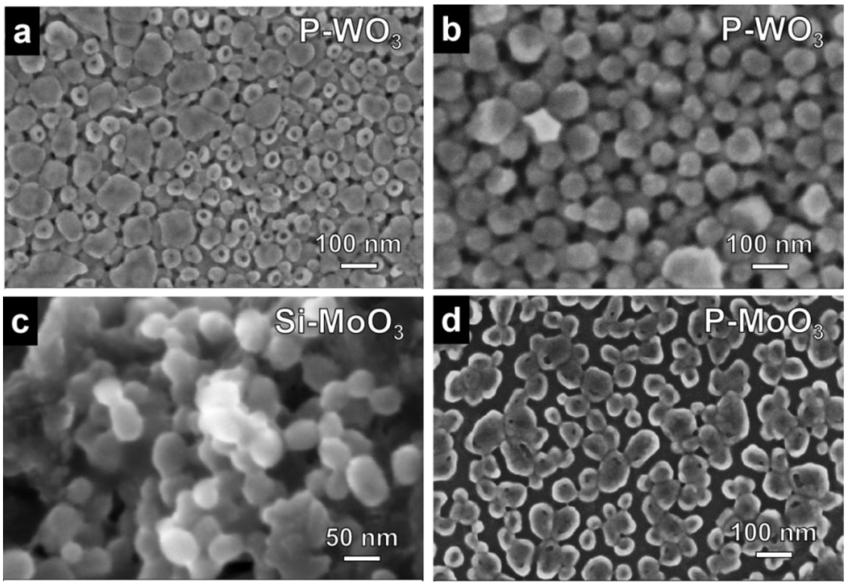

Figure S18. FESEM images of (a) P-WO_3 nanobowls, (b) P-WO_3 nanoparticles, (c) Si-MoO_3 nanobowls, (d) P-MoO_3 nanoparticles synthesized from co-assembly of PEO-*b*-PS with (a) $H_3PW_{12}O_{40}$, (b) $H_3PW_{12}O_{40}$, (c) $H_4SiMo_{12}O_{40}$ and (d) $H_3PMo_{12}O_{40}$ respectively in the THF/*n*-hexane mixed solvent.

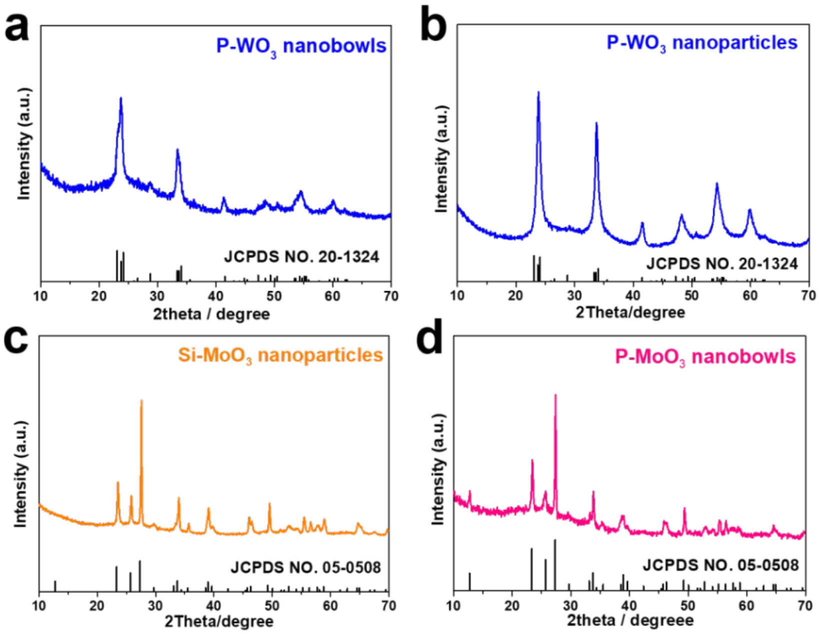

Figure S19. XRD patterns of the (a) P-WO_3 nanobowls, (b) P-WO_3 nanoparticles, (c) Si-MoO_3 nanoparticles and (d) P-MoO_3 nanobowls. The sharp diffraction peaks of the four samples indicates well-crystalline properties of the frameworks.

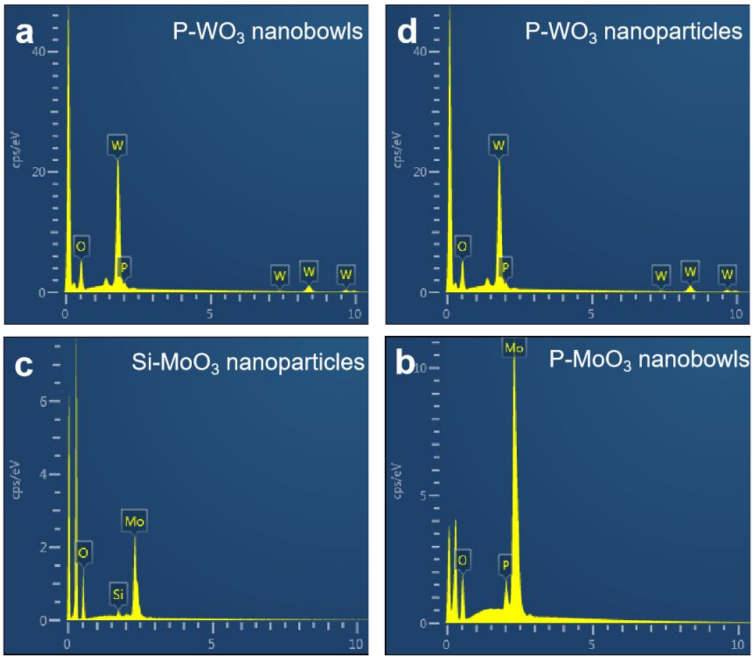

Figure S20. Energy dispersive spectrometer (EDS) patterns of the (a) P-WO_3 nanobowls, (b) P-WO_3 nanoparticles, (c) Si-MoO_3 nanoparticles and (d) P-MoO_3 nanobowls.

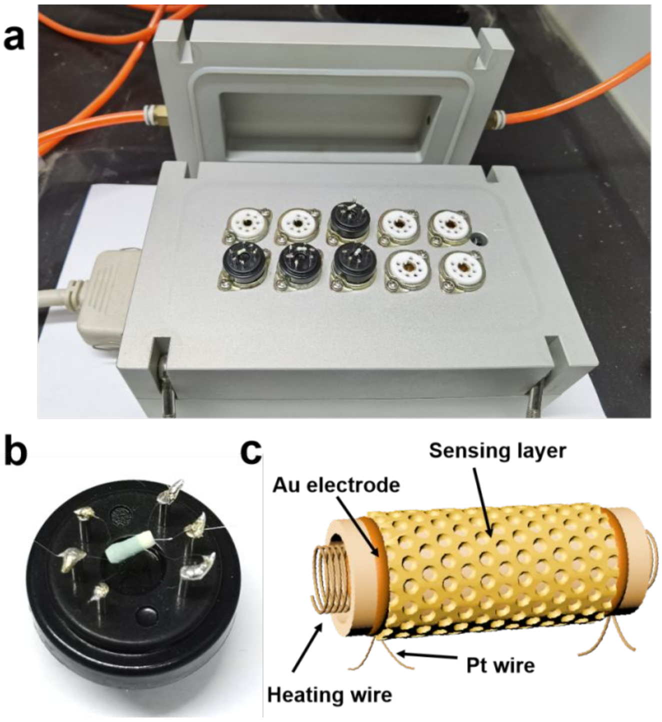

Figure S21. (a) Photograph of the gas sensing test device based on dynamic gas distribution system. (b) Photograph and (c) sketch of the structure of a side-heated gas sensor.

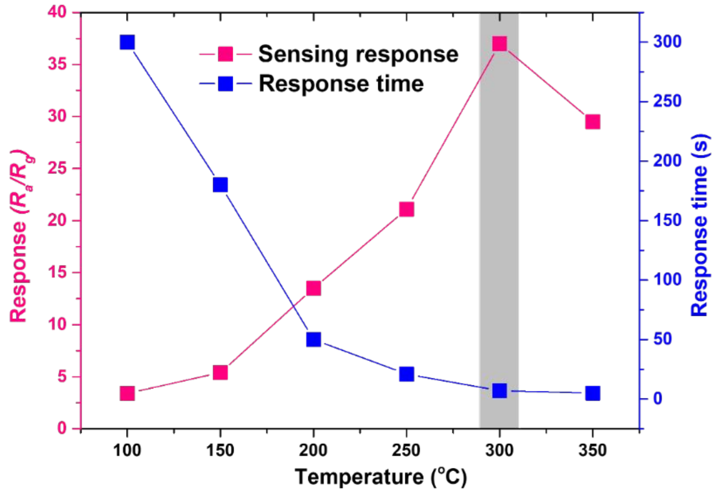

Figure S22. Sensing response and response time of the Si-WO_3 nanobowls-based sensor to 50 ppm of acetone at different working temperatures (100–350 ℃).

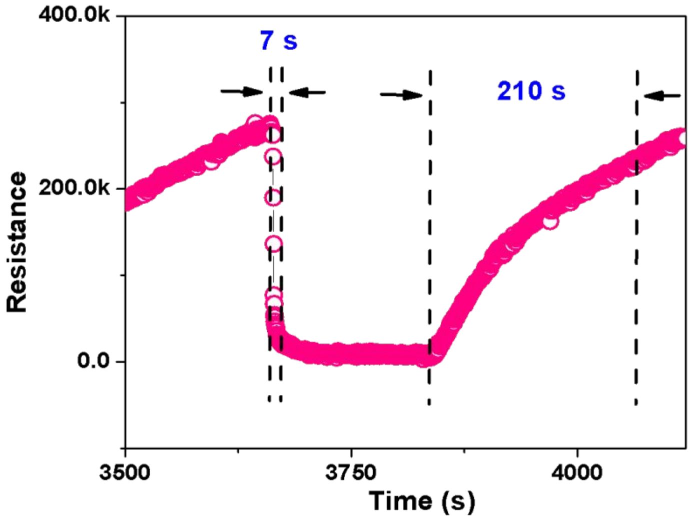

Figure S23. Response-recovery curve of the Si-WO_3 nanobowls based sensor to 50 ppm acetone at 300 °C.

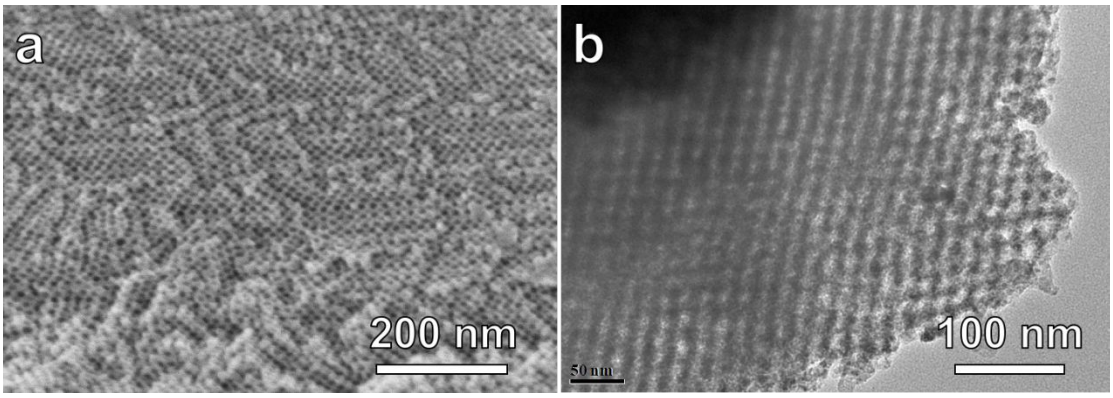

Figure S24. (a) SEM and (b) TEM images of the mesoporous WO_3 prepared by coassembly of PEO-*b*-PS and WCl_6 in THF.

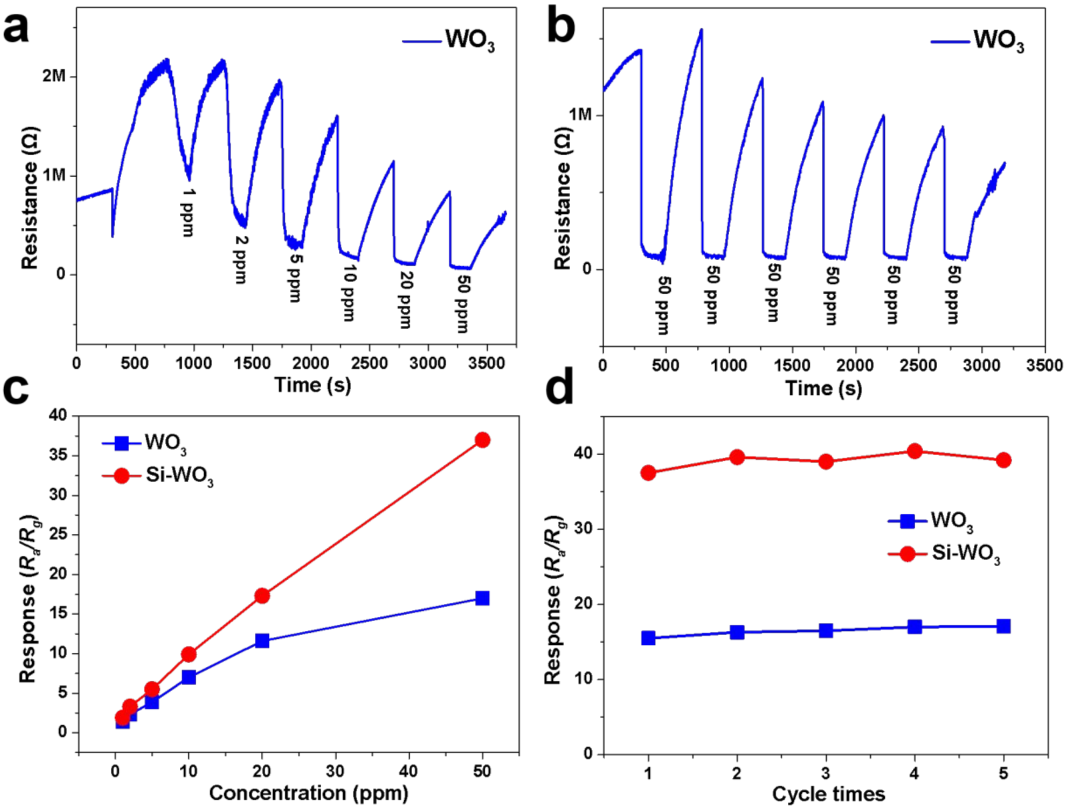

Figure S25. Response-recovery curves to (a) 1–50 ppm and (b) 50 ppm of acetone of the mesoporous WO_3 based senor; Sensing responses to (c) 1–50 ppm and (d) 50 ppm of acetone of the mesoporous WO_3 and Si-WO_3 nanobowls based sensors.

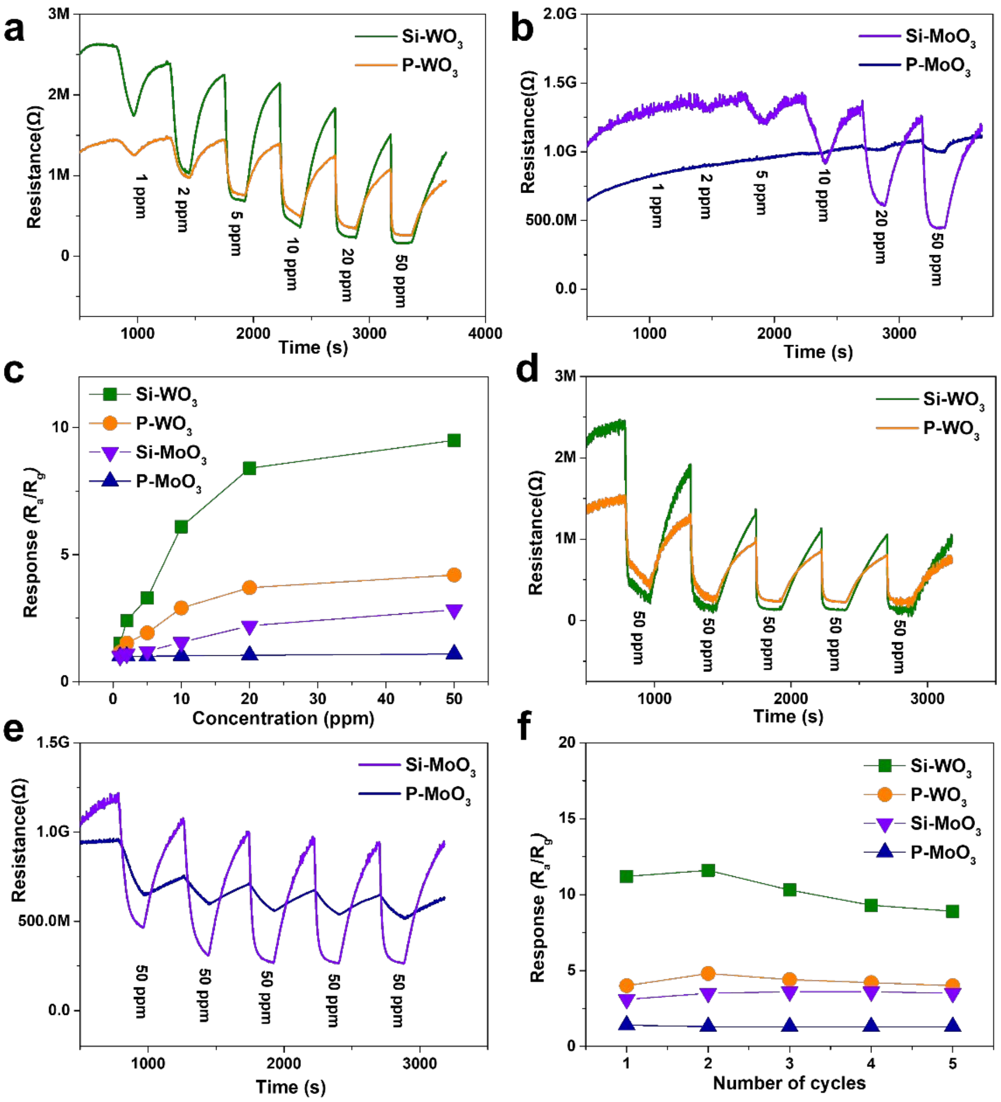

Figure S26. Gas sensing performances of the Si-WO_3, P-WO_3, Si-MoO_3 and P-MoO_3 nanomaterials-based sensor. (a, b) Dynamic response−recovery curves and (c) sensing responses to acetone of different concentration (1−50 ppm) at 300 °C. (d, e) Cycling performances and (f) and sensing responses to 50 ppm acetone at 300 °C.

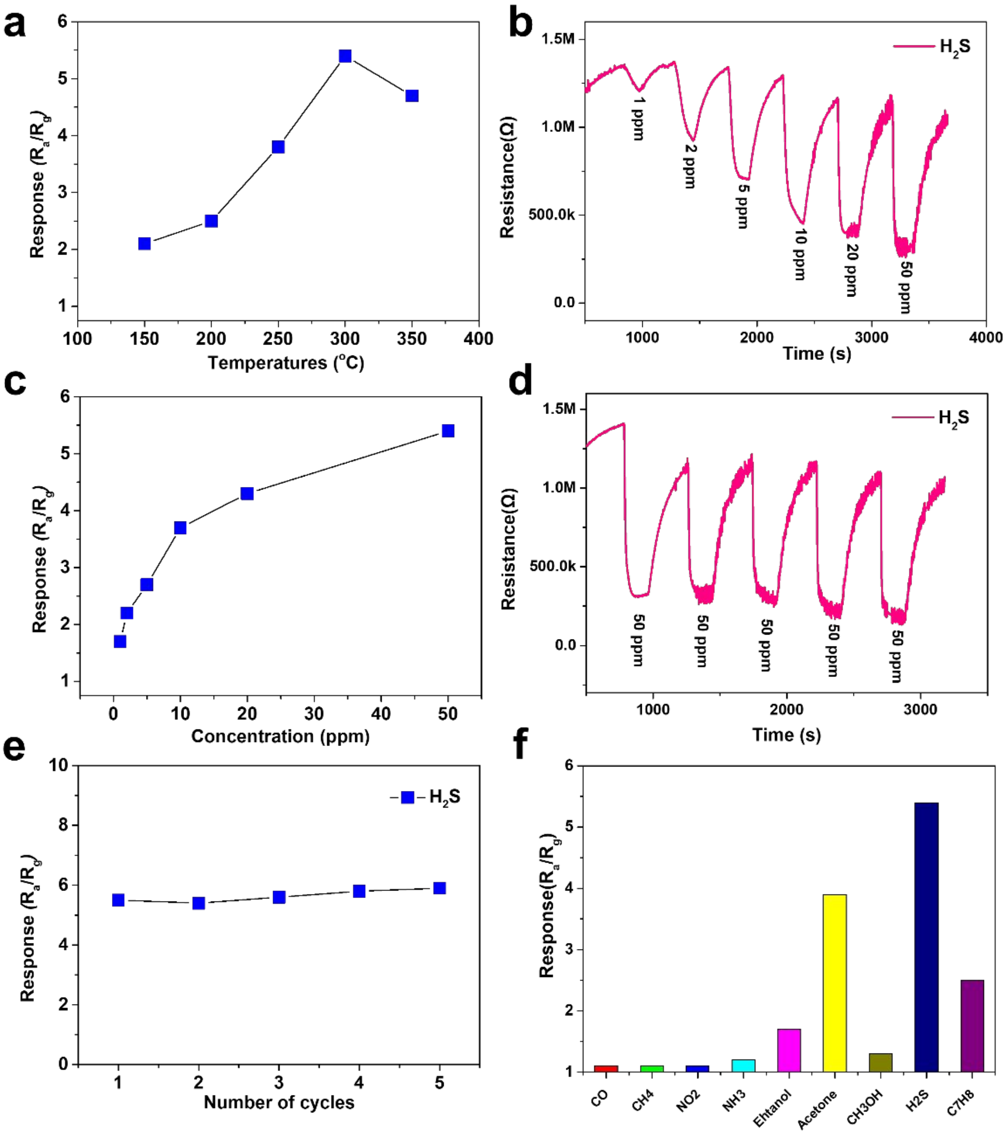

Figure S27. Gas sensing performances of the P-WO_3 nanoparticles-based sensor. (a) Sensing response to 50 ppm of H_2S at different working temperatures (150−350 °C). (b) Dynamic response−recovery curves and (c) sensing responses to H_2S of different concentration (1−50 ppm) at 300 °C. (d) Cycling tests to 50 ppm H_2S at 300 °C. (e) Sensing responses to 50 ppm of H_2S. (f) Sensing responses to 50 ppm of different kinds of gases.

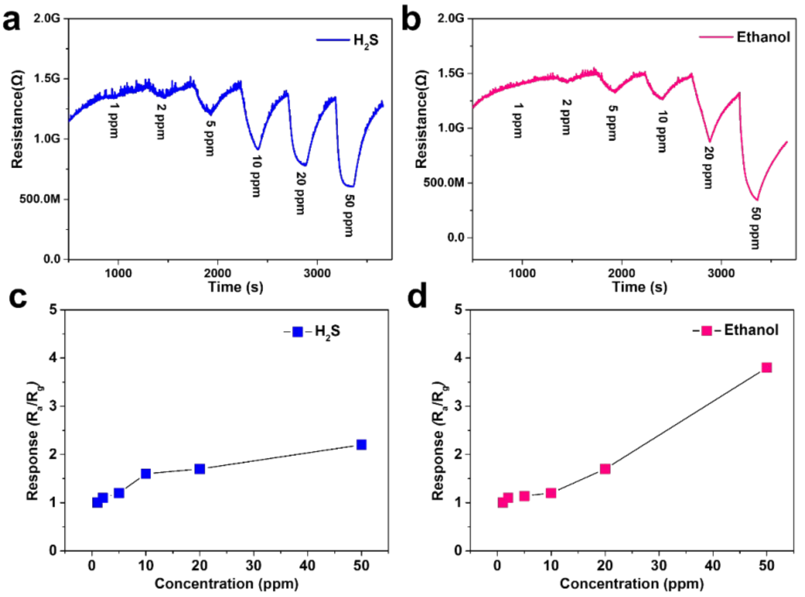

Figure S28. Gas sensing performances of the Si-MoO_3 nanoparticles-based sensor. Dynamic response−recovery curves to (a) H_2S and (b) ethanol of different concentration (1−50 ppm) at 300 °C. Sensing responses to (c) H_2S and (d) ethanol of different concentration (1−50 ppm).

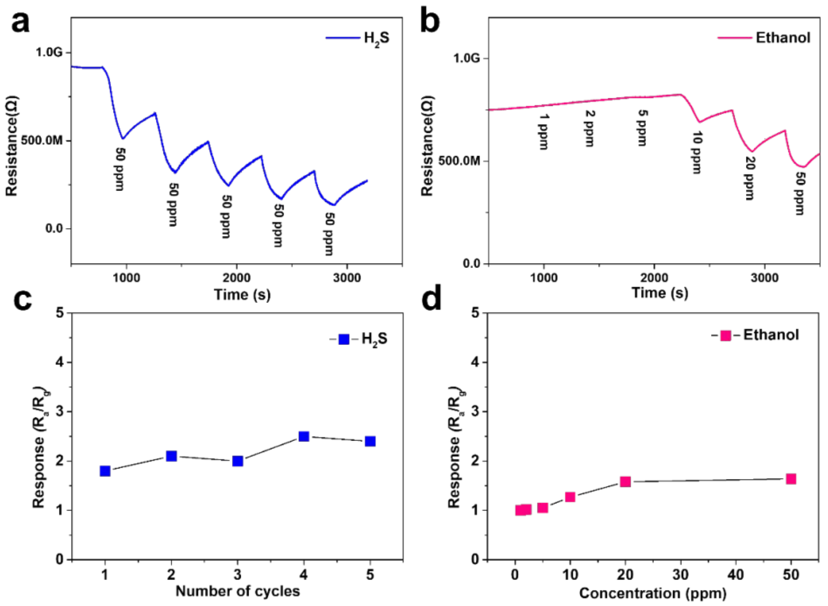

Figure S29. Gas sensing performances of the P-MoO_3 nanobowls-based sensor. (a) Cycling response−recovery curves to 50 ppm of H_2S at 300 °C. (b) Dynamic response−recovery curves to ethanol of different concentration (1−50 ppm) at 300 °C. (c) Sensing responses to 50 ppm of H_2S. (d) Sensing responses to ethanol of different concentration (1−50 ppm).

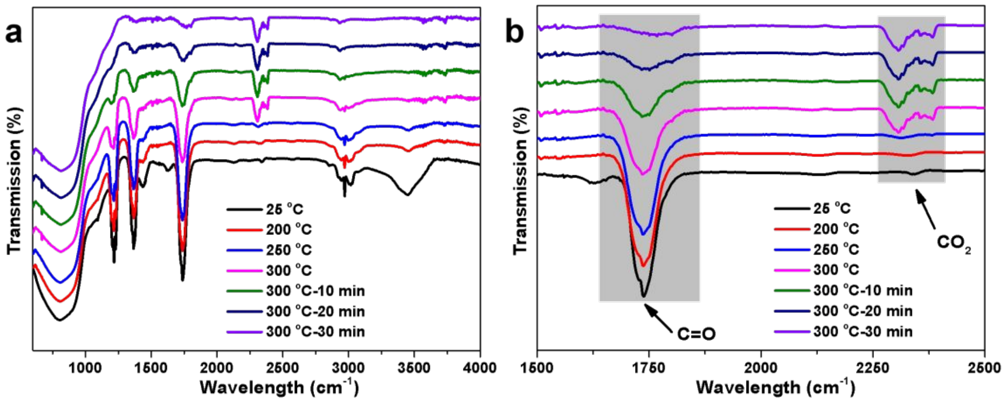

Figure S30. (a, b) In-situ Fourier transform infrared (*in-situ* FTIR) spectroscopy of the $Si\text{-}WO_3$ nanobowls reacting with acetone at different temperatures.

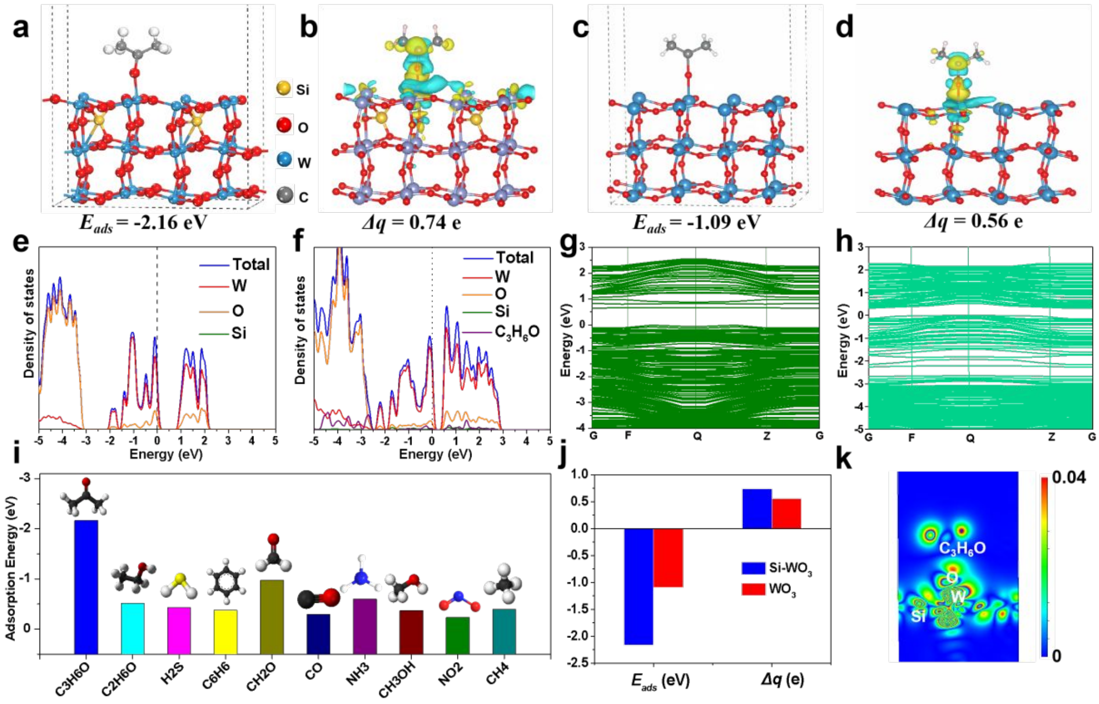

Figure S31. Optimized adsorption structures of the geometric binding configurations and charge distribution of the acetone molecule with the (020) plane of (a, b) $Si\text{-}WO_3$ and (c, d) WO_3; (e, f) Total and partial density of states (DOS) and the (g, h) calculated band structures of the $Si\text{-}WO_3$ before and after acetone adsorption; (i) Adsorption energies of different gases on the $Si\text{-}WO_3$ surface, a lower adsorption energy (further from zero) denotes a stronger adsorption; (j) Adsorption energy and differential charge density of acetone absorbed on $Si\text{-}WO_3$ and WO_3; (k) The charge density difference of the $Si\text{-}WO_3$ after acetone adsorption.

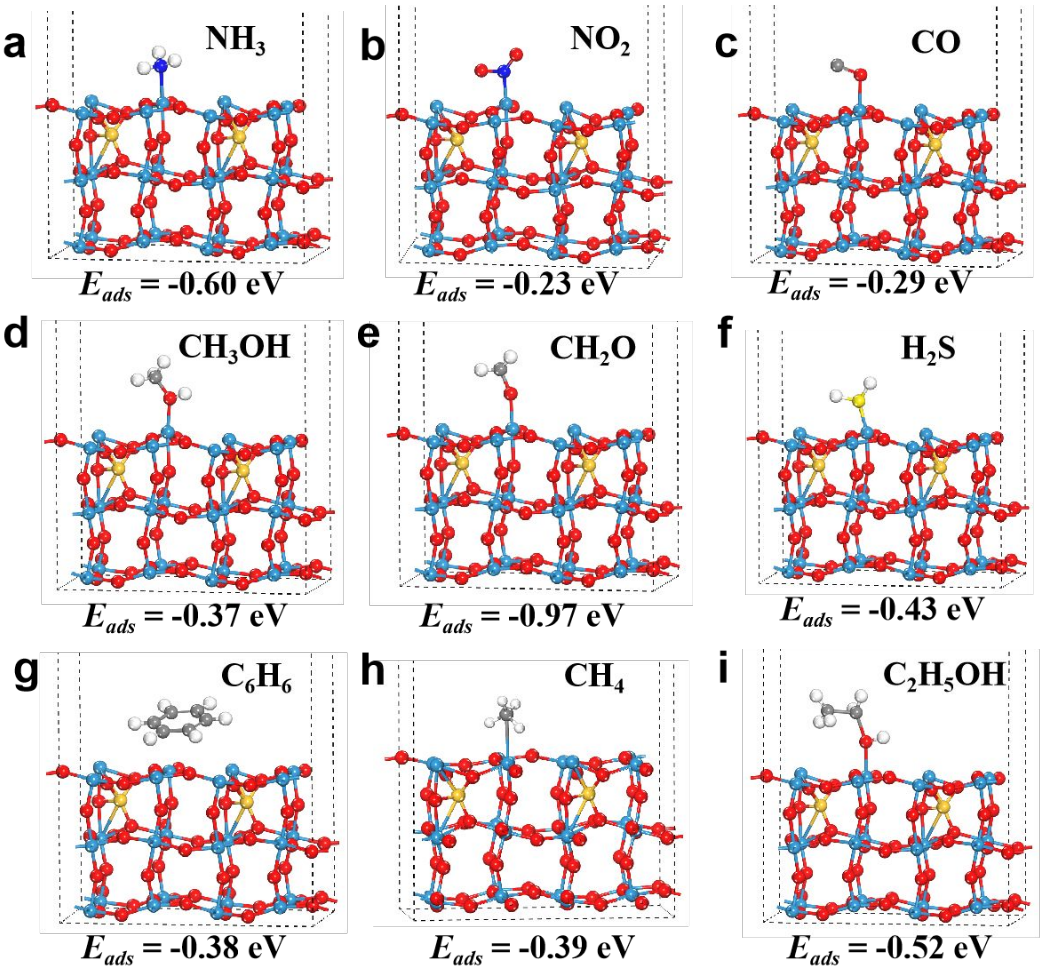

Figure S32. (a-i) Optimized adsorption structures of the geometric binding configurations and binding energies (E_{ads}) of different gas molecules with the (020) plane of Si-WO_3.

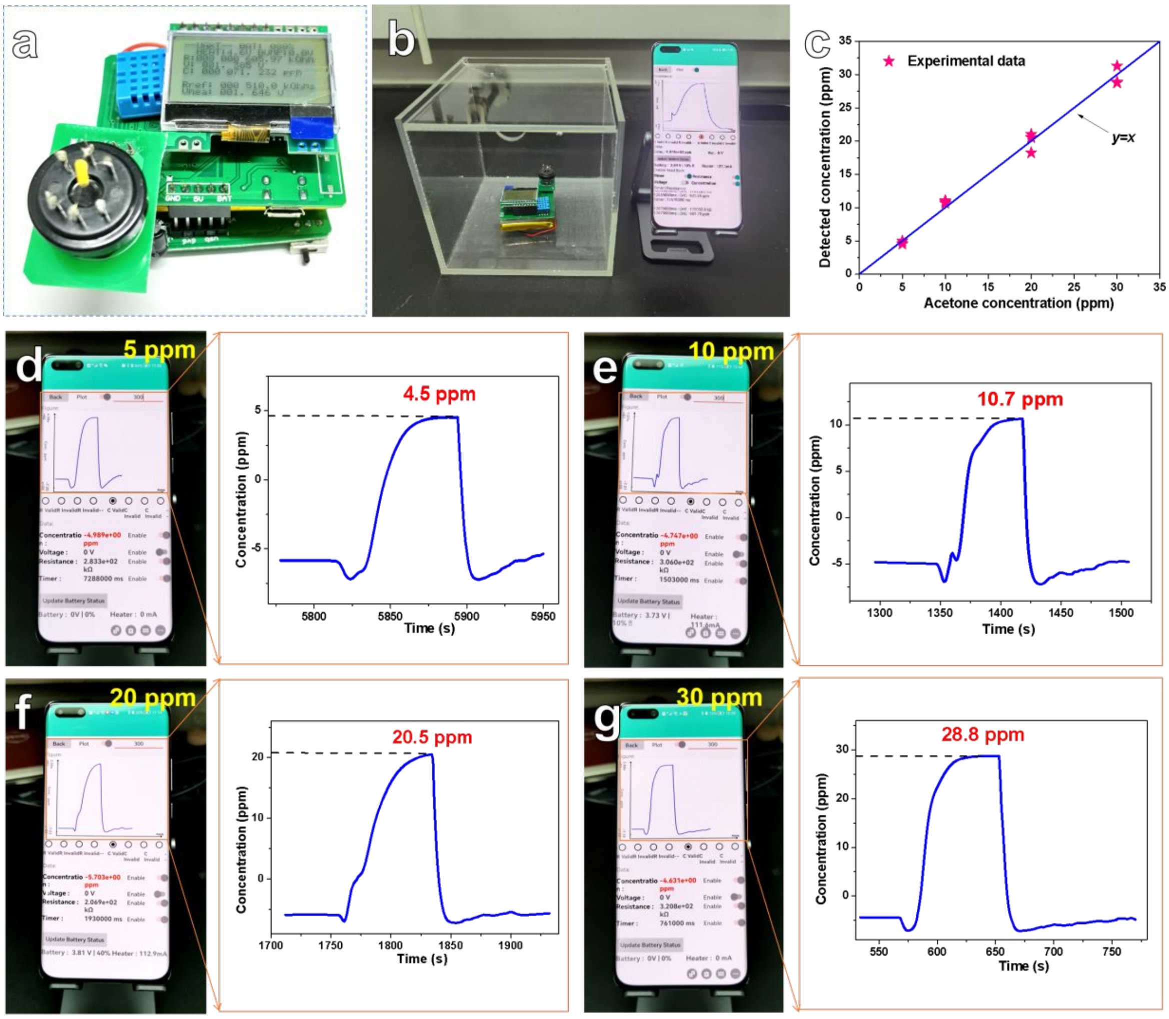

Figure S33. Photographs of the (a) integrated sensor module and (b) testing device, (c) the relationship of the real concentration of injected acetone and the concentration detected by the sensor; Real-time monitoring acetone of different concentration: (d) 5 ppm, (e) 10 ppm, (f) 20 ppm, (g) 30 ppm on the smart phone via Bluetooth communication.

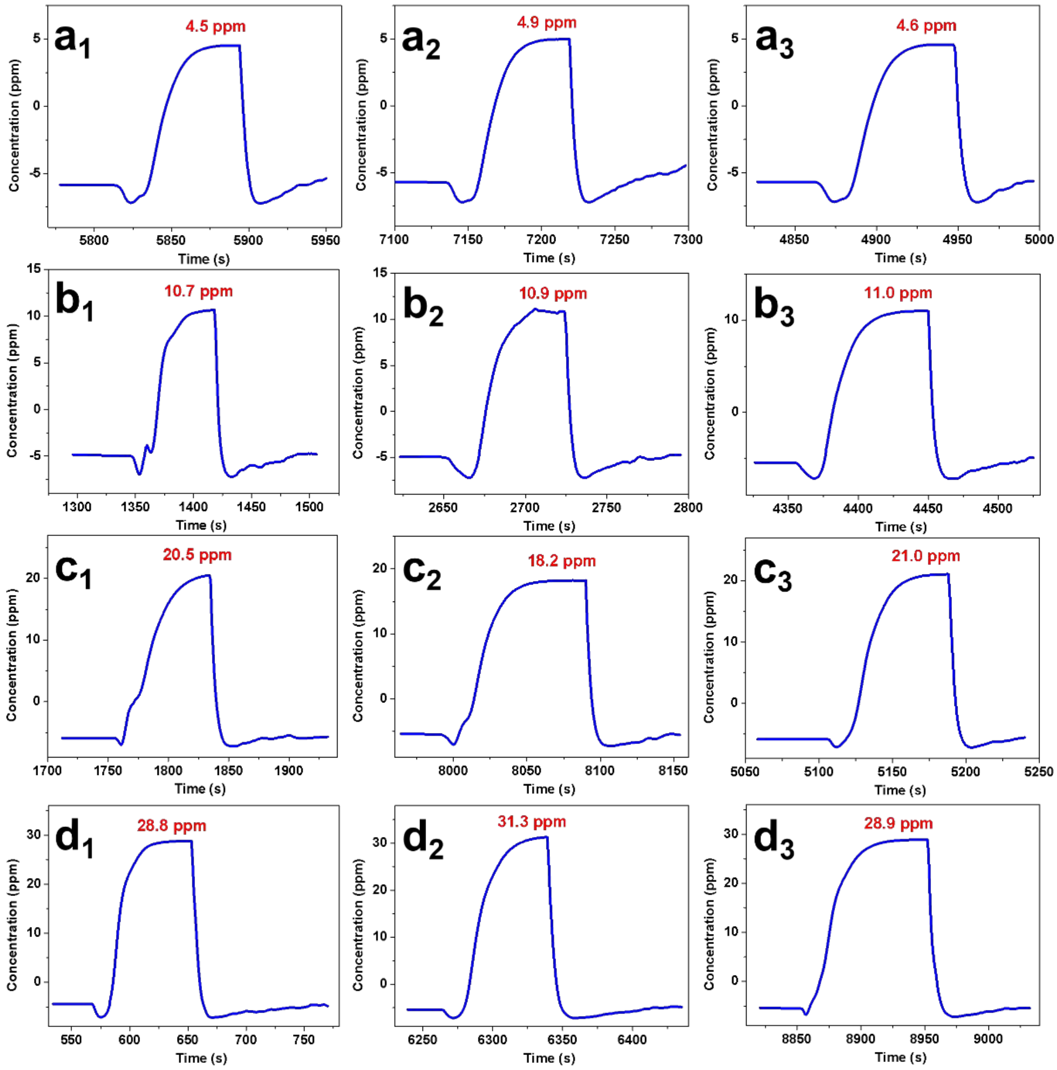

Figure S34. Repeating tests of the real-time monitoring acetone of different concentrations on a smart phone via Bluetooth communication. (a) 5 ppm, (b) 10 ppm, (c) 20 ppm, (d) 30 ppm.
